# Biotype and host relatedness influence the composition of bacterial microbiomes in *Schizaphis graminum* aphids

**DOI:** 10.3389/fmicb.2025.1614492

**Published:** 2025-07-30

**Authors:** Yan M. Crane, Charles F. Crane, Christian Webb, Brandon J. Schemerhorn

**Affiliations:** ^1^Crop Production and Pest Control Research Unit, USDA-ARS, West Lafayette, IN, United States; ^2^Department of Entomology, Purdue University, West Lafayette, IN, United States; ^3^Department of Botany and Plant Pathology, Purdue University, West Lafayette, IN, United States

**Keywords:** *Schizaphis graminum*, RNA-seq, microbiome, 16S rRNA, gut microflora, symbiont

## Abstract

**Introduction:**

The microbiome of greenbug aphid (*Schizaphis graminum* (Rondani)) was investigated in regard to greenbug biotype, collection date, host species, and host cultivar.

**Methods:**

DNA samples were collected from biotypes E and K feeding on 17 cultivars belonging to five host plant species, namely wheat, barley, rye, sorghum, and the goatgrass *Aegilops triuncialis*. Samples were taken immediately before infestation and two, four, and eight days thereafter. The V5-V7 hypervariable region of 16S rDNA was PCR amplified, Illumina sequenced, and aligned to a curated database of bacterial 16S rDNA sequences.

**Results and discussion:**

The almost universal intracellular endosymbiont of aphids, *Buchnera aphidicola*, comprised 78.24 to 99.99% of the read counts among samples, largely because of its high copy number of genomes per bacteroid. Abundant non-*Buchnera* genera included *Pseudomonas*, *Rhodanobacter*, *Massilia*, and *Enterobacter*. Read counts of eight of 78 examined genera were more than 90% restricted to a single replicate of a single treatment. Shannon entropy was highest in biotype K and on the barley host, but it did not vary significantly among dates post infestation. Unweighted UniFrac distances most significantly varied with biotype, host plant species, infestation time, and almost all of their interactions. Weighted UniFrac and Jaccard distances varied less significantly. By counts of differentially populated genera, the factors biotype, host plant species, infestation time, and host plant resistance genes to greenbug, were consecutively less important. Functional analysis with PICRUSt2 illustrated a diminution of respiratory electron transport and long-chain fatty acids in the Buchnera endosymbiont, reflecting adaptation to an intracellular environment.

## Introduction

Greenbug aphid [*Schizaphis graminum* (Rondani)] is a serious insect pest of small grain crops (Royer et al., 2015). It reduces yields by direct feeding and by transmitting plant-pathogenic viruses such as Cereal Yellow Dwarf Virus (CYDV). At the turn of the century, economic losses to greenbug exceeded $100 million per year to wheat and sorghum in the southern Great Plains of the United States (Eddleman et al., 1999). There exist several sources of resistance to greenbug, including the *Gb* gene in *Aegilops* (Zhu et al., 2005) and *Rsg1* and *Rsg2* in barley (Armstrong et al., 2016; Xu et al., 2023). Greenbug variants, traditionally termed biotypes, have been defined by their ability to infest different wheat and sorghum cultivars (Porter et al., 1997; Bowling et al., 1998). Currently 12 biotypes have been designated A through K and TX1 (Armstrong et al., 2016), but a thorough worldwide study of greenbug populations had not been conducted, and possibly many more biotypes remain to be discovered. Greenbug gene expression has been examined by RNA-seq in relation to biotype, time, and CYDV carrier status (Crane et al., 2023).

Aphid microbiomes exist in several sites: the gut, the specialized bacteriocyte cells, the hemolymph, the exterior cuticle, and the immediately local phylloplane. Each has its own conditions and potentially supports a distinct microflora. Almost all aphid taxa harbor *Buchnera aphidicola*, an intracellular mutualist bacterium that is restricted to bacteriocytes and is vertically transmitted from mother to offspring (Braendle et al., 2003). Other bacteria, such as *Hamiltonella defensa* (Gupta and Nair, 2020), *Rickettsia* sp. (Deng et al., 2019), and Candidatus *Fukatsuia symbiotica* (Michalik et al., 2014; Manzano-Marín et al., 2023) have also been found to inhabit bacteriocytes, sometimes with *Buchnera* and sometimes separately. The roles of these endosymbionts in aphid nutrition, reproduction, and defense against fungi and parasitoids have been extensively investigated (Csorba et al., 2024). However, the gut is where the most generalist and diverse microflora is to be expected and where a diverse community of apparent commensals exists (Guyomar et al., 2018). Here the microflora is in direct contact with the aphid’s nutritionally deficient diet of plant sap, and here the properties of the host plant can directly affect the competition among microbial taxa (Wilson and Duncan, 2015). These properties include carbohydrate and protein concentration, amounts of antimicrobial peptides and phenolics, pH, and levels of minerals and vitamins (Casteel and Hansen, 2014; Gupta and Nair, 2020; Yun et al., 2014).

Amplification of hypervariable regions within 16S rRNA genes is a popular and effective way to census bacterial populations (Johnson et al., 2019), since the 16S locus is universally present in bacteria (Geng et al., 2022; Haro et al., 2021), highly conserved primer sequences exist (Haro et al., 2021; Thijs et al., 2017), large databases of 16S amplicon sequences are available, the method does not depend upon gene expression, and there are software packages (e.g., QIIME2) specifically designed to process such sequences. However, the method is not perfect, since repeated amplification cycles can magnify the effect of slight variation in primer binding and elongation efficiency, and since some bacterial genomes are present in high copy numbers per cell (notably including *Buchnera aphidicola*; Komaki and Ishikawa, 1999). Amplicon sequencing will miss any taxa whose 16S hypervariable regions cannot be amplified for any reason.

Previous studies of aphid microbiomes vary in the amount of variation reported within species and among localities. Only a subset deals with host plant as an experimental variable. In general, reports that include rare taxa find more variation among localities or host plants than reports that exclude rare taxa. This reflects the generally high fraction of *Buchnera* among read counts. When *Buchnera* comprises 70% or more of reads, there is less opportunity for rare taxa to reach a 1% threshold of abundance that is often used to filter out possible contaminants.

On the basis of Bray–Curtis dissimilarities among 70 samples obtained from 44 host aphid species, McLean et al. (2019) concluded that aphid microbiomes were more correlated with aphid species than with host plant, and this correlation was highly significant for both secondary symbionts and non-symbionts. They found a lesser correlation between aphid microbiomes and host plant habit (tree versus herb) that could reflect the taxonomic relationships of the aphids that infest each. McLean et al. (2019) also noted that the abundance and diversity of non-symbiotic microbes were less in aphids than in insects with richer diets. Microbiome correlation to aphid taxon is necessarily higher in aphid taxa that seasonally alternate between hosts. For example, Wu et al. (2018) reported that 171 microbial operational taxonomic units (OTUs) were shared between summer and winter generations of *Schlechtendalia chinensis*, while 12 OTUs were restricted to winter and 11 were restricted to summer. The winter and summer host plants differ radically: a moss (*Plagiomnium maximoviczii*) versus sumac (*Rhus chinensis*).

Using field-collected specimens, Gallo-Franco et al. (2019) found that weighted and unweighted UniFrac distances of microbiomes of *Aphis gossypii* and *Myzus persicae* were more clustered together within aphid species than within host *Capsicum* species. Also with field-collected specimens, Fakhour et al. (2018) found greater similarity of non-*Buchnera* microflora within each of five aphid species than within collection localities. Zepeda-Paulo et al. (2018) found variation among field collections in secondary symbiont frequency in *Sitobion avenae* but essentially no variation among field collections of *Rhopalosiphum padi*, where *Buchnera* accounted for at least 98.5% of read counts. Xu et al. (2021) found little effect of collection locale or host plant on alpha diversity or Bray–Curtis dissimilarity in 92 samples of *Myzus persicae* field collected from 16 host plant families and 30 sites across China. However, *Buchnera aphidicola* dominated all of the samples, and seven secondary symbionts accounted for most of the remaining counts of taxa that exceeded 1% of the total in any one sample. Each of these seven genera monopolized the non-*Buchnera* counts in at least one sample. Therefore, the taxonomic composition of the *Myzus* microbiome could reflect host plant variation or aphid genetics or location history.

Ma et al. (2021) compared *Aphis gossypii* field-collected on hibiscus and pomegranate with samples of 50 individuals transferred from hibiscus to cotton, muskmelon, and cucumber and then propagated for 10 generations. Because all of these samples were taken from naturally established populations in the field, they were potentially genetically heterogenous. They found that the microbiomes’ taxonomic composition diverged after transfer to the second host plant species and that the Shannon index (within-sample diversity) increased after transfer to muskmelon and cucumber. Thus, in this case a change in diet did affect the microbiome. It is noteworthy that Ma et al. (2021) did not restrict analysis to taxa with at least 1% abundance in at least one sample, whereas Xu et al. (2021) did. If bacterial ploidy increases during the evolution of obligate endosymbiosis as it did in *Buchnera* (Komaki and Ishikawa, 1999), it is possible that the combined counts of *Buchnera* and the newly evolved co-obligate endosymbiont would push the maximum count of any other taxon below 1%.

He et al. (2021) conducted a similar experiment with *Myzus persicae*, which was transferred from Chinese cabbage (the control species) to eggplant, pepper, or tobacco and allowed to feed and reproduce for 14 days. The mean Shannon index increased approximately three-fold on pepper relative to eggplant, tobacco, or Chinese cabbage. Bray–Curtis dissimilarities clustered together for aphids on Chinese cabbage. The taxonomic composition shifted from abundant *Buchnera* on Chinese cabbage to abundant *Pseudomonas* on eggplant and tobacco. Pepper had about half as much *Pseudomonas* as *Buchnera*. Quantitative PCR showed that the absolute abundance of *Buchnera* decreased on eggplant and tobacco and remained about the same on pepper as in Chinese cabbage. The ratio of total bacterial to aphid DNA was approximately constant over the four diets.

Two studies considered the effect of diet on the same aphid population. Ikuze et al. (2024) reported family-level frequencies of bacteria from samples of the same *Melanaphis sacchari* population placed on *Melanaphis* resistant and susceptible sorghum inbreds. The microbiome was sampled seven and 14 days later. While the report is sparse with details and has inconsistencies with respect to the families reported versus collection time and host plant resistance, it appears that the resistant and susceptible diets did not significantly affect alpha diversity according to the Chao1 index, which increased from day 7 to day 14 on both diets. The pattern of bacterial family frequencies was similar between diets, but the susceptible diet favored Sphingomonadaceae and Pseudomonadaceae at the expense of Burkholderiaceae and Rhizobiaceae. Cassone et al. (2015) looked specifically at *Buchnera* transcription and titer in *Aphis glycines* feeding on healthy soybean plants and soybean plants infected with bean pod mottle virus, which aphids do not transmit. They found reduced transcription and titer on the infected plants, which were associated with reduced aphid fecundity. *Buchnera* transcription recovered when aphids were moved from infected to healthy soybean plants. They noted that the reduced aphid fitness also occurred on soybean infected with two other viruses and attributed the reduction to the dietary quality of infected soybean phloem.

Because of greenbug’s economic importance, multiple resistance genes have been sought and found in landraces and wild relatives of wheat, barley, and sorghum. Among others, these genes include *Gb1* through *Gb6* in wheat (Porter et al., 1994), *Gbx1* and *Gbz* in wheat wild relative *Aegilops triuncialis* (Zhu et al., 2005), *Rsg1* and *Rsg2* in barley (Xu et al., 2023), and *SgR1* in sorghum (Zhang et al., 2024). The mechanisms of action of these resistance genes are generally unknown, although *SgR1* encodes a leucine-rich repeat receptor-like protein that is expressed in transgenic *Arabidopsis thaliana* exposed to aphid feeding (Zhang et al., 2024). Their net effect is to inhibit greenbug growth and reproduction, and in doing so they potentially affect the composition and activity of the greenbug microbiome.

Although the type strain of *Buchnera aphidicola* was chosen from *Schizaphis graminum* (Munson et al., 1991) and early genomic studies on *B. aphidicola* were conducted on the *S. graminum* strain (Lai and Baumann, 1992; Munson et al., 1993; Rauhbakhsh and Baumann, 1995), the greenbug microbiome seems not to have been reported to date. Here we have investigated how host plant species and cultivar affect the bacterial flora of two greenbug biotypes, E and K, over time. The hosts were 17 cultivars from five species, sorghum, wheat, rye, barley and *Aegilops triuncialis*. Read counts from the PCR-amplified V5-V7 region of 16S rDNA served as a proxy for bacterial populations.

The experiment addressed several hypotheses: (1) that the microbiome responds to host greenbug genetics, here biotype; (2) that the microbiome similarity is related to phylogenetic relationship of the host plants; (3) that the microbiome responds to host-plant resistance genes; and (4) that the microbiome responds to elapsed time. The null hypothesis in each case is that the microbiome does not respond to that factor. The experiment was not designed to elucidate the mechanism by which each factor influenced the microbiome, in that the host plant was not analyzed for any of the plausible mechanisms (nutrition, physical impediments, antibiosis) that could influence the greenbug microbiome, and the greenbugs themselves were not weighed or measured.

## Materials and methods

### Sample sources and collection

Greenbug biotypes E and K were obtained from Dr. Gary Puterka of the USDA-ARS Wheat, Peanut and Other Field Crops Research Unit in Stillwater, Oklahoma. A single apterous female of biotype E founded the population of biotype E, and a single apterous female of biotype K founded the population of biotype K. Both parthenogenetic populations were increased in isolation to hundreds of individuals on wheat cv. “Newton” prior to the experiment. There were four cycles of increase where adult aphids were placed on “Newton” and allowed to give birth for two days before the adults were removed. Eight days elapsed from the start of the fourth cycle to the beginning of the experiment. Therefore, the populations were somewhat synchronized by age. The experiment was conducted in two growth chambers, one at 25°C on a 12-h day and 12-h night cycle with 60% humidity for sorghum and the other at 20°C on a 14-h day and 8-h night cycle with 50% humidity for the other four host plant species. There were 17 cultivars among five host plant species as listed in Table 1. Each combination of biotype and host cultivar was isolated in a separate cage containing four potted plants. Without vernalization, host plants did not flower or undergo visible phase change during the experiment and remained free of visible disease.

**Table 1:** Host list with susceptibility to biotypes E and K as listed by Armstrong et al. (2016).

ID	Species	Scientific name	Cultivar (resistance gene)	E	K
W1	Wheat	*Triticum aestivum L*.	Newton	S	S
W2	Wheat	*Triticum aestivum L*.	Custer	S	S
W3	Wheat	*Triticum aestivum L*.	Amigo (Gb2)	S	S
W4	Wheat	*Triticum aestivum L*.	Largo (Gb3)	R	R
W5	Wheat	*Triticum aestivum L*.	C117882 (Gb5)	R	R
W6	Wheat	*Triticum aestivum L*.	GRS 1201 (Gb6)	R	R
S1	Sorghum	*Sorghum bicolor L*.	TX7000	S	S
S2	Sorghum	*Sorghum bicolor L*.	TX2737	S	S
S3	Sorghum	*Sorghum bicolor L*.	TX2783	R	S
S4	Sorghum	*Sorghum bicolor L*.	DZhugara Belaya = PI 550607	NT	NT
B1	Barley	*Hordeum vulgare L*.	Wintermalt	S	S
B2	Barley	*Hordeum vulgare L*.	Jao (Rsg2)	NT	NT
B3	Barley	*Hordeum vulgare L*.	Post 90 (Rsg1)	R	R
R1	Rye	*Secale cereale L*.	Elbon	S	S
R2	Rye	*Secale cereale L*.	Insave F.A. (Gb2 and Gb6)	R	R
A1	Aegilops	*Aegilops triuncialis L*.	TA 1675 (Gbz)	NT	NT
A2	Aegilops	*Aegilops triuncialis L*.	TA 1695 (Gbx1)	NT	NT

S, susceptible; R, resistant; NT, not tested.

The initial, day zero sampling was taken from aphids on “Newton” wheat. On day zero, approximately 15 (10 to 20) individual greenbugs were placed on each of four plants of each cultivar. Greenbugs were sampled from three of the four plants 2, 4, and 8 days after placement. Each plant constituted a biological replicate. Most samples consisted of 10 to 15 greenbugs, but samples ranged from two to 20 individuals. We attempted to equalize the mass of greenbugs used for library construction by sampling more individuals of small greenbugs and fewer individuals of large greenbugs. For each plant, the collected aphids were wrapped together in labeled aluminum foil, dropped into liquid nitrogen, and then stored at −80°C. There were 312 samples in all.

### Preparation for sequencing

Total greenbug DNA was extracted by using the DNeasy Blood and Tissue Kit for DNA Isolation (Qiagen, Germantown, MD) following its instructions. The hypervariable V5–V7 region of 16S rDNA was amplified using the 799F (AACMGGATTAGATACCCKG) and 1193R (ACGTCATCCCCACCTTCC) primers (Haro et al., 2021). The MyFi^™^ DNA polymerase kit (Bioline, Taunton, MA) was used for PCR with a protocol modified from Haro et al. (2021). Initial denaturation at 95° for 5 min preceded 30 cycles of 95°C for 60s, 55°C for 45 s and extension at 72°C for 60 s. Final elongation was at 72°C for 8 min followed by holding at 4°C. The expectedly 394 bp PCR product was confirmed by electrophoresis in 1.5% agarose gels with SYBR safe DNA stain (Thermo Fisher Scientific, Waltham, MA).

### Sequencing and data analysis

16S rDNA was sequenced using the Illumina MiSeq platform. After quality filtering, reads were processed through the software pipeline of RTL Genomics (Lubbock, TX), which is detailed at http://www.rtlgenomics.com/docs/Data_Analysis_Methodology.pdf. This pipeline invoked programs for clustering at 97% identity, denoising, eliminating chimeric reads, alignment to a company-curated subset of NCBI rRNA accessions, and referring operational taxonomic units to taxa. Beyond that, OTU read counts were aggregated by genus, host plant species and cultivar, aphid biotype, and sampling date, for barplot display with ggplot2 in R. The pipeline normalized all samples to the lowest read count of 301,562 by using scaling with ranked subsampling (Gomes et al., 2024). A rarefaction survey was performed at intervals of 25,000 counts. It removed OTUs that mapped to *Ralstonia*, which has been recognized as a common contaminant in DNA extraction kits (Salter et al., 2014). It also removed unidentifiable OTUs and rare OTUs that comprised less than 0.01% of the total reads in that particular sample. The resulting filtered OTUs were subjected to standard tests of taxonomic diversity in R packages vegan (Oksanen et al., 2025) and phyloseq (McMurdie and Holmes, 2013): Shannon entropy (Shannon, 1948), Hill1 diversity (Hill, 1973), weighted (Lozupone et al., 2006) and unweighted UniFrac (Lozupone and Knight, 2005) with principal coordinates analysis. An analysis of variance (ANOVA) was performed with read counts grouped by greenbug biotype, host plant species, host cultivar, and time from infestation. Pairwise comparisons of read counts for host plant species, infestation durations, and combinations of biotype and host plant species, were tested with Tukey’s Honest Significant Difference test (Tukey, 1949). ANOVA and Tukey’s HSD were also performed for the same groups with Shannon entropy and Hill1 diversity. PERMANOVA (R program Adonis by McMurdie and Holmes, 2013) was performed with weighted UniFrac, unweighted UniFrac, and Jaccard dissimilarities (Jaccard, 1901) among the same groups used in ANOVA. Differential “expression” (population) was tested with ancombc (Lin and Peddada, 2020) with zero cut = 0.80, struc zero = false, and neg lb = false.

The effect of greenbug-resistance genes was analyzed separately from the effects of biotype, cultivar, and collection date. The resistance genotype was known for 13 cultivars as listed in Table 1, and it was the same for biotypes E and K in 12 of them. For these 12 cultivars, the reads obtained from RTL Genomics were imported into QIIME2 (Bolyen et al., 2019) and denoised with dada2 (Callahan et al., 2016). By using custom perl scripts, the resulting representative reads (amplified sequence variants, ASV) were extracted to 20 fasta files and simultaneously aligned to SILVA 138.2 (Quast et al., 2013; Yilmaz et al., 2014) with blastn (Camacho et al., 2009). A taxon was assigned to each ASV on the basis of closest blast hit. The frequency table of ASVs and the taxonomic assignment were imported back into QIIME 2, where the ASVs were collapsed to genera. The table of frequencies grouped by taxa was then subjected to ancombc. A second run of ancombc was performed on the frequency table after removing the *Buchnera* counts. A perl script collated the ensuing log2 fold-changes and *p*-values into a table.

Physiological functions of the microbiome were inferred from taxon abundance with PICRUSt2 (Douglas et al., 2020). PICRUSt2 was run on the representative sequences (amplified sequence variants, ASV) output by DADA2 (Callahan et al., 2016) in QIIME2 (Bolyen et al., 2019). There were separate runs for *Buchnera* ASV, non-*Buchnera* ASV, and all ASV together. Perl scripts were written to fill out the pathway names and rank them by reported pathway activity so that *Buchnera* could be compared to the rest of the microbiome.

## Results

**Figure 1:**
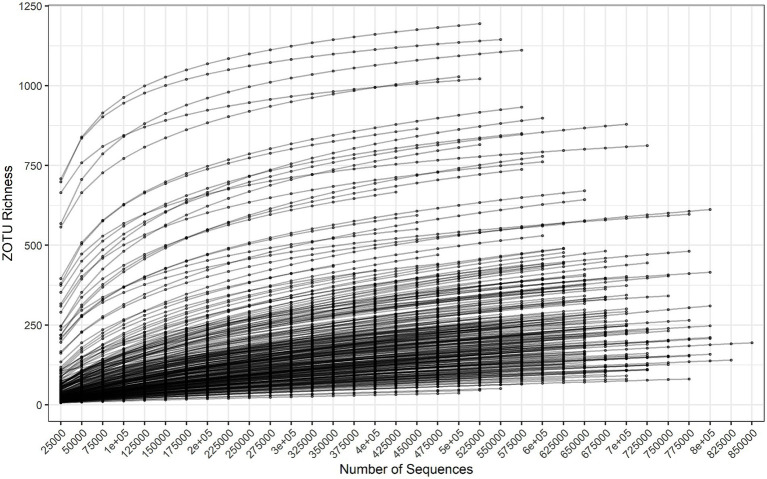
Rarefaction survey of OTU richness, subsampling from 25,000 to 850,000 reads in increments of 25,000 reads.

Read counts per sample varied from 301,562 to 853,907, with a mean of 603,858 and median of 608,703. Figure 1 is a rarefaction plot generated by repeated subsampling of quality-filtered read counts and identification by alignment to the in-house database of 16S rRNA sequences. The counts were used before normalization to 301,562 counts across samples, so each curve is limited to its total read count. The least diverse samples saturated more than the more diverse samples, but no sample exhausted its own taxonomic variation. At least 1,150 operational taxonomic units (OTUs) were observed in the most diverse samples. Overall there were 1,511 OTUs representing 78 genera.

**Figure 2:**
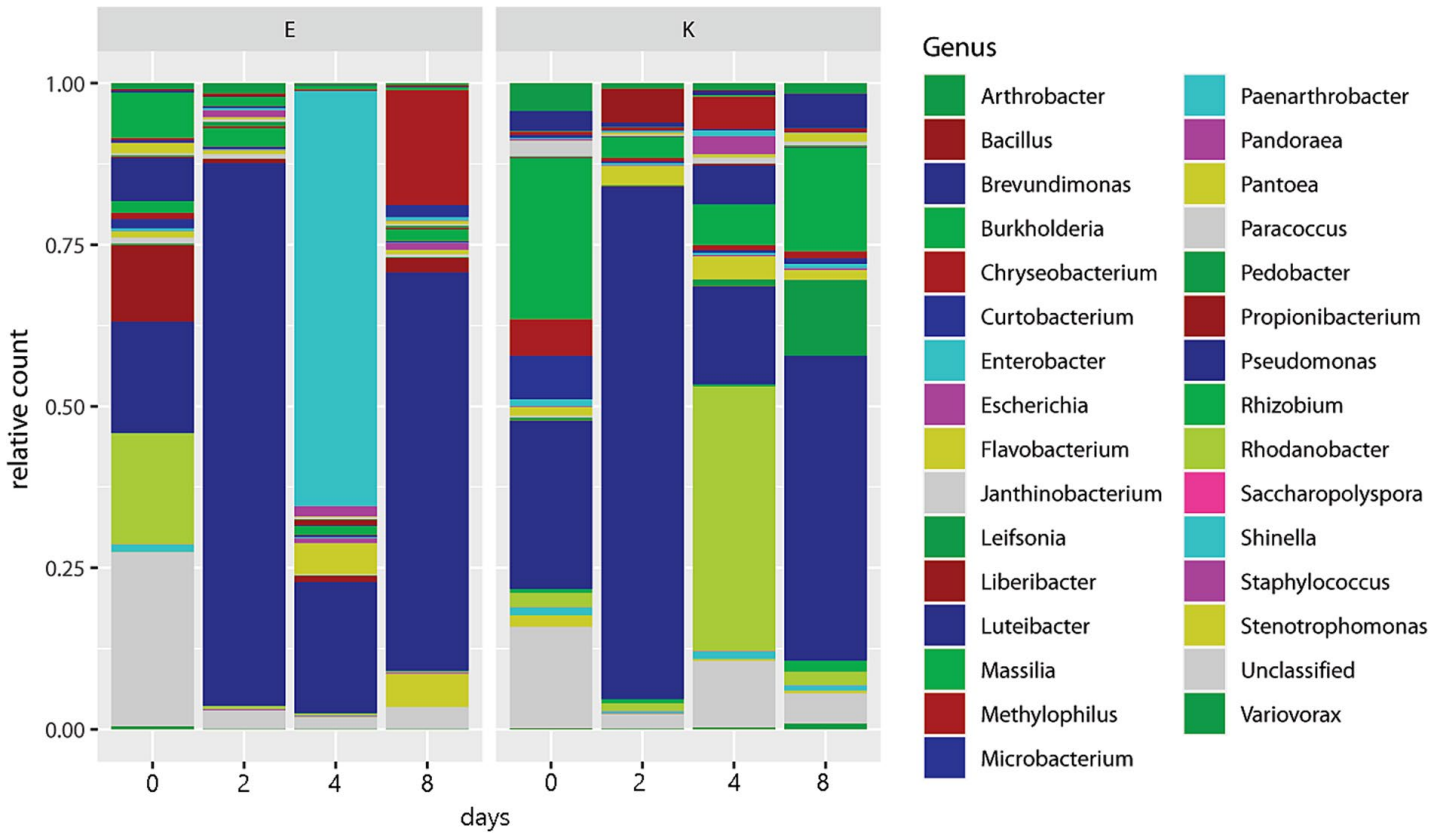
Barplot of 30 most abundant genera sorted by greenbug biotype and days post infestation.

Only 15 out of 78 identified genera had over 10,000 total counts (Table 2). *Buchnera aphidicola* dominated the counts, outnumbering second-place *Pseudomonas* by a factor of 196 and third-place *Rhodanobacter* by a factor of 840. *Buchnera* was also by far the most evenly distributed genus among the conditions and replicates, being the only genus with less than 10% of its total counts in any single condition or replicate. At the other extreme, several genera, such as *Deinococcus*, *Sphingobium*, and *Luteibacter*, were almost entirely restricted to a single replicate despite having thousands of counts (Table 2). With the exception of one unidentified species of *Serratia*, no OTUs mapped to genera of secondary endosymbionts in aphids (*Hamiltonella*, *Regiella*, *Arsenophonus*, *Candidatus Fukatsuia*, *Rickettsia*, *Rickettsiella*, and *Wolbachia*). To display the relative abundance of other genera, stacked barplots were prepared with *Buchnera* counts excluded. Figure 2 gives the fractions of the 30 most abundant genera other than *Buchnera* in relation to time and aphid biotype over all hosts. *Pseudomonas* peaked in both biotypes on days 2 and 8, while *Rhodanobacter* was prominent on day 0 in biotype E and day 4 in biotype K. *Enterobacter* was most common in biotype E on day 4, and the related genus *Escherichia* was at its maximum in both biotypes on day 4. In biotype K, *Chryseobacterium*, *Luteibacter*, and *Pantoea* peaked on day 4, *Massilia* peaked on days 4 and 8, while *Bacillus* peaked on day 2 and *Pedobacter* peaked on day 8. *Chryseobacterium* was also high in biotype E on day 4. Unclassified genera, which were identified only to a higher taxonomic level, were most abundant in both biotypes on day 0.

**Table 2:** Fraction of a taxon’s total counts restricted to a single treatment (combination of a single biotype, cultivar, and timepoint) or replicate within a treatment.

Genus	Treatment	Replicate	Total count
*Deinococcus*	0.9954	0.9948	11,419
*Sphingobium*	0.9821	0.9821	1,061
*Luteibacter*	0.9632	0.9628	15,257
*Citrobacter*	0.9438	0.9424	5,430
*Xanthomonas*	0.9410	0.9384	2,304
*Paraburkholderia*	0.9274	0.9274	124
*Rhodanobacter*	0.9200	0.9189	109,638
*Klebsiella*	0.9099	0.9080	1,631
*Humibacter*	0.8922	0.8660	306
*Enterobacter*	0.8906	0.8890	43,554
*Herbaspirillum*	0.8754	0.8754	666
*Bacillus*	0.8488	0.8476	10,288
*Phenylobacterium*	0.8403	0.8403	238
*Serratia*	0.8286	0.8286	426
*Dyella*	0.8266	0.8252	738
*Acinetobacter*	0.7849	0.7849	172
*Kosakonia*	0.7448	0.7448	968
*Telluria*	0.7326	0.7209	86
*Devosia*	0.7320	0.7295	403
*Pseudolabrys*	0.7273	0.6591	88
*Escherichia*	0.6821	0.6807	10,034
*Novosphingobium*	0.6517	0.5493	2,159
*Leifsonia*	0.6376	0.5486	2,111
*Acidovorax*	0.6295	0.6235	672
*Brevundimonas*	0.6074	0.6070	11,276
*Paenibacillus*	0.6010	0.5984	381
*Chryseobacterium*	0.5509	0.5503	22,244
*Stenotrophomonas*	0.5471	0.5471	4,005
*Bordetella*	0.5463	0.5458	2,248
*Caulobacter*	0.5315	0.5280	286
*Ramlibacter*	0.5312	0.5312	160
*Asticcacaulis*	0.5312	0.5312	1,299
*Lysinimonas*	0.5280	0.4800	125
*Pedobacter*	0.4979	0.4976	21,383
*Erwinia*	0.4918	0.4895	1,287
*Achromobacter*	0.4688	0.4688	689
*Thermomonas*	0.4613	0.4611	4,875
*Ulvibacter*	0.4609	0.4609	256
Unclassified	0.4374	0.4109	47,141
*Janthinobacterium*	0.4258	0.4254	4,403
*Lysobacter*	0.4164	0.4164	305
*Hydrogenophaga*	0.4016	0.4016	381
*Pantoea*	0.3995	0.3939	21,089
*Sphingomonas*	0.3905	0.3342	1,493
*Staphylococcus*	0.3857	0.3831	770
*Mesorhizobium*	0.3787	0.3577	478
*Saccharopolyspora*	0.3738	0.3641	206
*Curtobacterium*	0.3658	0.3658	2,258
*Cutibacterium*	0.3593	0.3593	462
*Ktedonobacter*	0.3561	0.3485	132
*Rhizobium*	0.3549	0.3502	4,477
*Luteimonas*	0.3485	0.3485	264
*Peredibacter*	0.3299	0.3260	776
*Sinorhizobium*	0.3153	0.3114	517
*Flavobacterium*	0.3146	0.3128	5,467
*Shinella*	0.3110	0.3106	4,994
*Propionibacterium*	0.3090	0.3057	3,278
*Burkholderia*	0.3018	0.2966	4,798
*Arachidicoccus*	0.2986	0.2986	278
*Arthrobacter*	0.2880	0.2139	10,824
*Bradyrhizobium*	0.2796	0.1935	93
*Methylobacillus*	0.2704	0.1415	318
*Variovorax*	0.2693	0.2550	2,310
*Pseudacidovorax*	0.2664	0.2617	214
*Paracoccus*	0.2652	0.1973	2,017
*Pseudomonas*	0.2609	0.2126	468,875
*Massilia*	0.2554	0.2551	56,338
*Mycobacterium*	0.2500	0.1304	92
*Pelomonas*	0.2365	0.2365	148
*Pandoraea*	0.2102	0.2096	1,889
*Solimonas*	0.2032	0.2032	1,604
*Microbacterium*	0.1998	0.1864	4,334
*Nocardioides*	0.1953	0.1523	256
*Paenarthrobacter*	0.1915	0.1785	2,992
*Methylophilus*	0.1795	0.1776	4,876
*Liberibacter*	0.1696	0.1627	2,299
*Caenibaculum*	0.1229	0.1208	480
*Buchnera*	0.0098	0.0033	92,054,787

**Figure 3:**
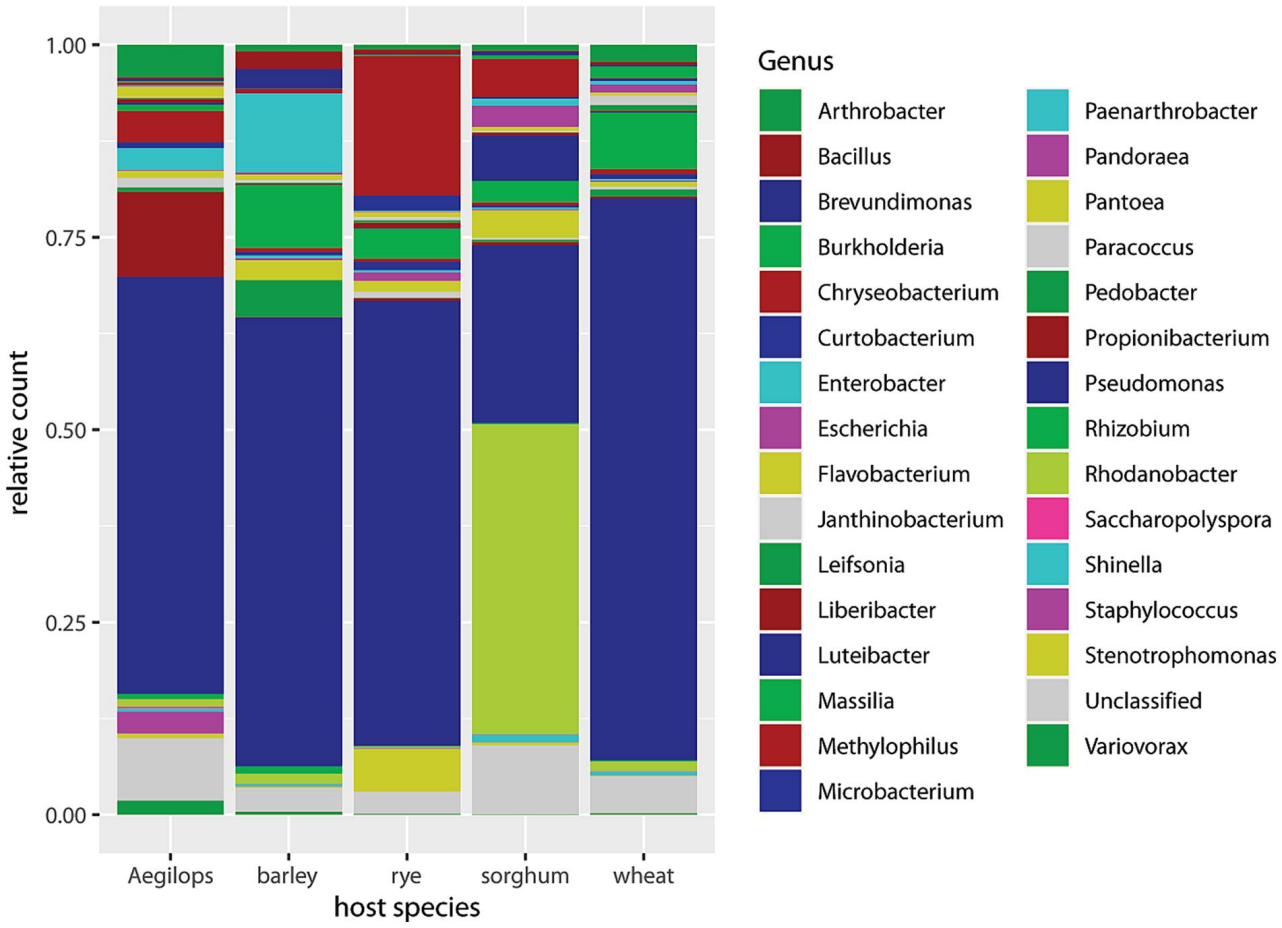
Barplot of 30 most abundant genera sorted by host plant species.

Figure 3 displays the relative abundances by host plant species, summing over timepoints, cultivars, and aphid biotypes. Sorghum differed from the other four species in having less *Pseudomonas* and more *Rhodanobacter*. *Luteibacter*, *Chryseobacterium*, *Pantoea*, *Massilia*, *Escherichia*, and “unclassified” were also prominent in sorghum. The other four host plant species are all related and belong to the tribe Triticeae, and their non-*Buchnera* microbiomes were dominated by *Pseudomonas*. In barley, *Enterobacter*, *Massilia*, *Pedobacter*, *Pantoea*, *Brevundimonas*, and *Bacillus* were consecutively less prominent. In rye, *Chryseobacterium*, *Stenotrophomonas*, *Massilia*, *Curtobacterium*, and *Pantoea* were consecutively less abundant. In *Aegilops*, *Propionibacterium*, *Arthrobacter*, *Methylophilus*, *Staphylococcus*, and *Paenarthrobacter* were consecutively less abundant. In wheat, *Massilia*, *Arthrobacter*, *Burkholderia*, *Rhodanobacter*, and *Janthinobacterium* were consecutively less abundant.

**Figure 4:**
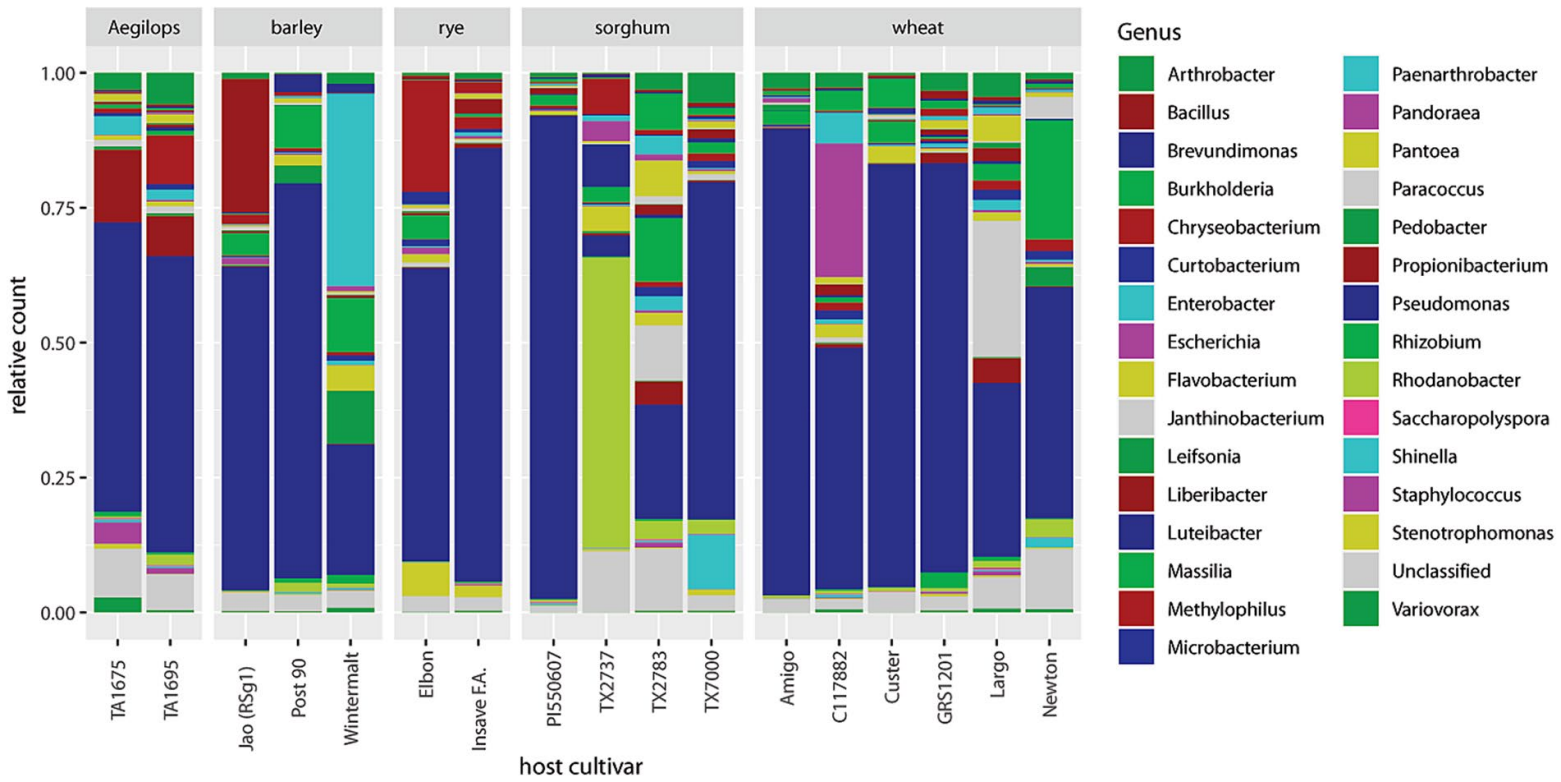
Barplot of 30 most abundant genera sorted by host plant species and cultivar.

Relative abundances of non-*Buchnera* genera are barplotted by cultivar in Figure 4. *Pseudomonas* dominated in 13 of 17 cultivars. Four cultivars (Wintermalt barley, TX2737 and TX2783 sorghum, and Largo wheat) stood out because of their relative deficiency of *Pseudomonas* and varying abundance of other genera. In Wintermalt, *Enterobacter*, *Massilia*, *Pedobacter*, and *Pantoea* were prominent. In TX2737, *Rhodanobacter*, *Luteibacter*, and *Chryseobacterium* were prominent. In TX2783, prominent genera included *Massilia*, *Paracoccus*, *Burkholderia*, and *Flavobacterium*. In Largo, *Paracoccus*, *Flavobacterium*, and *Propionibacterium* were relatively prominent. There was no correlation of low abundance of *Pseudomonas* to greenbug biotype resistance among these four cultivars as noted by Armstrong et al. (2016).

Statistical significance of variation in genus counts was appraised with ancombc (Lin and Peddada, 2020). Comparisons of the two biotypes, the four timepoints, and the five host plant species are reported in Table 3, which lists beta coefficients (the slope of the plot of log of taxon abundance divided by days after infestation), their standard error, Holm-adjusted *p*-values, and a statistic W that is the quotient of the beta value divided by its standard error. A positive beta slope for biotypes indicates up-population in biotype K over biotype E. A positive beta slope for timepoint indicates up-population at a later collection date versus day zero. A positive beta slope for host plant species indicates minimum population in *Aegilops*. Only three of the genera listed in the biotype section of Table 3 (*Rhodanobacter*, *Luteibacter*, and *Pedobacter*) are prominent in any bar of Figure 2. In the time section of Table 3, only *Bacillus* is prominent in a bar (biotype K on day 2) in Figure 2. In the species section of Table 3, four of the five identified genera were also prominent in Figure 3.

**Table 3:** Differentially populated genera by biotype, time, and host plant species.

Genus	Log fold-change	Std. error	*W*	Adj. *p*-value
By biotype				
*Methylophilus*	0.941	0.0728	12.930	1.399 × 10^−36^
*Solimonas*	0.734	0.0601	12.215	1.162 × 10^−32^
*Arthrobacter*	0.960	0.0934	10.279	3.866 × 10^−23^
*Rhizobium*	0.563	0.0576	9.767	6.699 × 10^−21^
*Paracoccus*	−0.763	0.0853	−8.946	1.555 × 10^−17^
*Paenarthrobacter*	0.641	0.0727	8.819	4.743 × 10^−17^
*Microbacterium*	0.544	0.0681	7.989	5.431 × 10^−14^
*Shinella*	0.454	0.0573	7.934	8.268 × 10^−14^
*Propionibacterium*	−0.758	0.0973	−7.797	2.404 × 10^−13^
Unclassified *Actinomycetia*	−0.525	0.0676	−7.771	2.882 × 10^−13^
*Brevundimonas*	0.482	0.0650	7.415	4.371 × 10^−12^
Unclassified *Alphaproteobacteria*	−0.534	0.0829	−6.441	4.164 × 10^−9^
*Burkholderia*	−0.465	0.0757	−6.139	2.821 × 10^−8^
*Variovorax*	0.395	0.0654	6.040	5.092 × 10^−8^
*Sphingomonas*	0.236	0.0440	5.361	2.641 × 10^−6^
*Pedobacter*	0.333	0.0624	5.347	2.767 × 10^−6^
*Caenibaculum*	−0.230	0.0484	−4.753	6.021 × 10^−5^
*Rhodanobacter*	0.355	0.0899	3.953	2.240 × 10^−3^
*Luteibacter*	0.256	0.0663	3.859	3.187 × 10^−3^
*Staphylococcus*	−0.287	0.0797	−3.605	8.439 × 10^−3^
*Mesorhizobium*	0.114	0.0319	3.566	9.428 × 10^−3^
*Cutibacterium*	−0.240	0.0698	−3.437	1.472 × 10^−2^
By time				
Unclassified *Saccharibacteria*	0.801	0.2145	3.734	8.683 × 10^−3^
*Bacillus*	1.094	0.2958	3.699	9.750 × 10^−3^
*Flavobacterium*	1.213	0.3427	3.540	1.758 × 10^−2^
By host plant species				
*Enterobacter*	1.524	0.2755	5.532	1.457 × 10^−6^
*Brevundimonas*	0.818	0.2134	3.835	5.645 × 10^−3^
Unclassified *Betaproteobacteria*	0.457	0.1196	3.819	5.902 × 10^−3^
Unclassified *Proteobacteria*	0.466	0.1324	3.518	1.867 × 10^−2^
*Janthinobacterium*	0.455	0.1332	3.419	2.634 × 10^−2^
*Massilia*	0.864	0.2527	3.420	2.634 × 10^−2^
*Pantoea*	0.861	0.2653	3.246	4.688 × 10^−2^

With respect to greenbug resistance genes, when ancombc (Lin and Peddada, 2020) was run with a frequency feature table of 479 genera that included *Buchnera*, there were seven genera with unadjusted *p*-values less than 0.01 as listed in Table 4. None of these was significant even at *p* < 0.10 after Benjamini–Hochberg correction for multiple testing. Log_2_ fold-changes from susceptible to resistant were modest, ranging from −0.32 for *Chryseobacterium* to 0.52 for *Massilia*. Positive and negative log_2_ fold-changes were split 274 to 205, so there was an excess of genera that became somewhat more abundant in resistant plants at the expense of *Buchnera*. Excluding *Buchnera* changed the result almost imperceptibly in Table 4.

**Table 4:** The 10 most nearly significantly differentially populated genera for resistant versus susceptible host cultivars.

Genus	With *Buchnera*			Without *Buchnera*		
	logFC	*p*-value	*q*-value	logFC	*p*-value	*q*-value
Erwinia	0.196	0.002	0.568	0.195	0.002	0.568
Curtobacterium	0.419	0.002	0.568	0.419	0.002	0.568
Sphingopyxis	0.162	0.005	0.568	0.162	0.005	0.568
Bacillus	0.295	0.006	0.568	0.295	0.006	0.568
Hyphomicrobium	0.195	0.006	0.568	0.195	0.006	0.568
Colwellia	0.110	0.008	0.590	0.110	0.008	0.591
Kurthia	0.234	0.009	0.590	0.234	0.009	0.591
Solimicrobium	0.191	0.012	0.737	0.191	0.012	0.736
Fusobacterium	0.280	0.016	0.855	0.280	0.016	0.856
Cupriavidus	0.089	0.022	0.980	0.089	0.022	0.977

**Figure 5:**
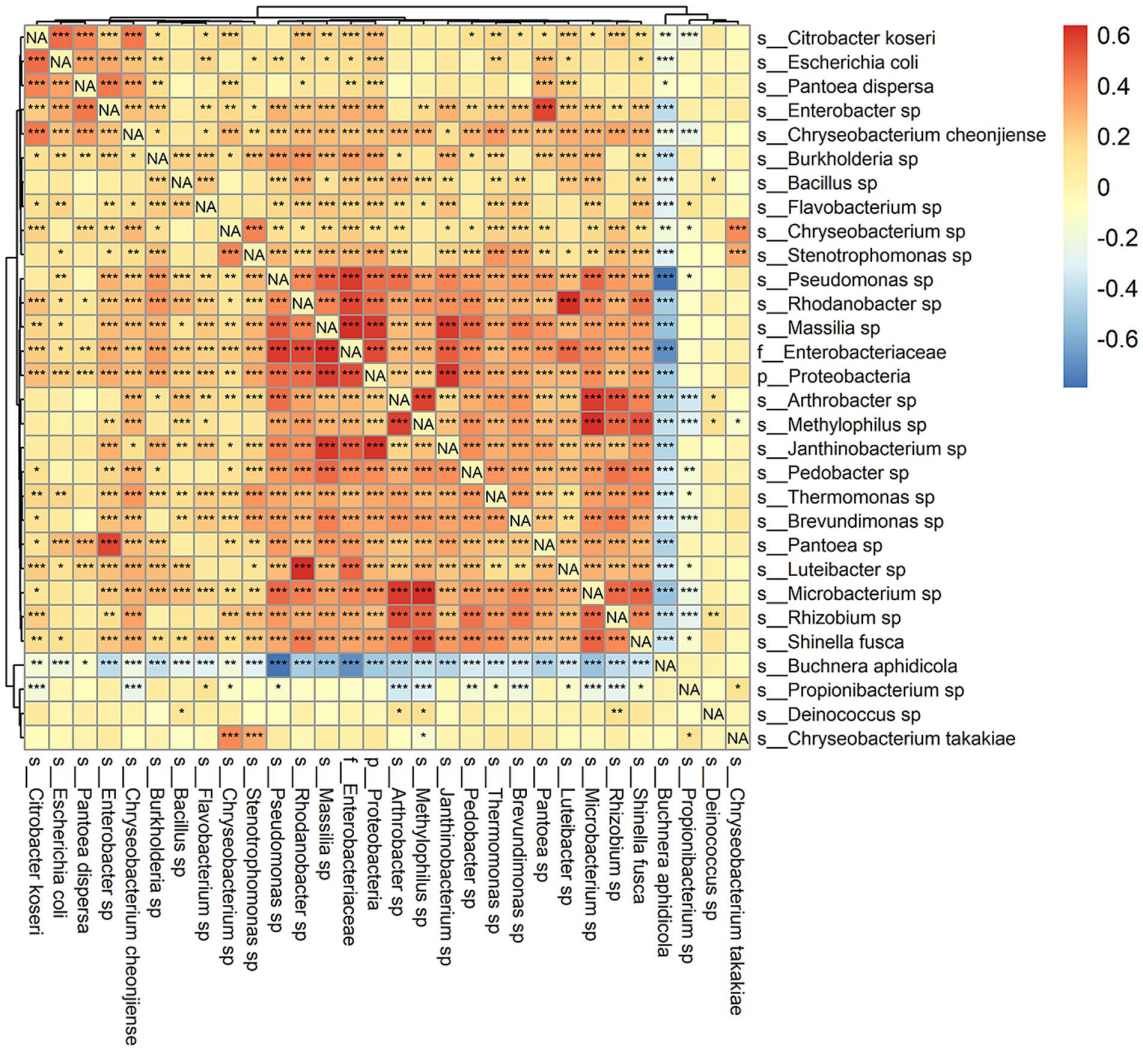
Spearman’s correlation of observed relative abundance among species. More positive values indicate greater strength of association. Significance testing was also performed for each pair of taxa, and asterisks indicate: ^*^p < 0.05, ^**^p < 0.01, and ^***^p < 0.001.

Correlations among frequencies of bacterial species appear in Figure 5. *Buchnera* was negatively correlated with all other taxa except *Pantoea dispersa*, *Propionibacterium* sp., *Deinococcus* sp., and *Chryseobacterium takakiae*. The negative correlations were mostly significant at *p* < 0.001 and were strongest for *Pseudomonas* (the most abundant genus other than *Buchnera*) and an unspecified genus of Enterobacteriaceae. Mutually positive correlations were conspicuously clustered in a group of 16 taxa in the lower right quadrant of Figure 5 and a second group of five taxa at the upper left corner of Figure 5. There was an isolated strongly positive correlation between species of *Enterobacter* and *Pantoea*. While correlations outside of the two groups were weaker, many were still significant at *p* < 0.001. Only *Deinococcus* sp. and *Chryseobacterium takakiae* were not strongly correlated with other taxa overall.

**Figure 6:**
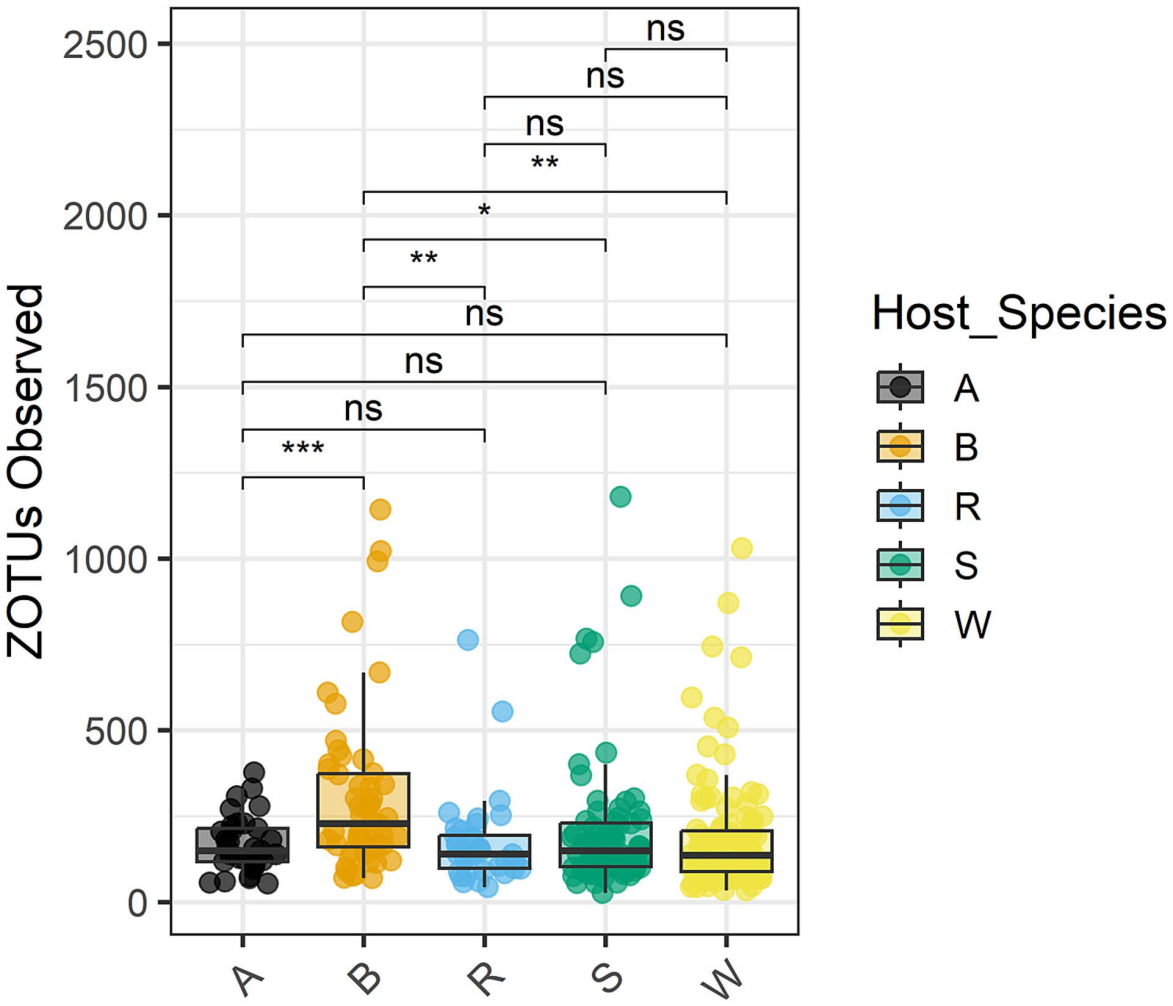
Zero-radius OTUs observed within the total microbiome data, grouped and colored by host plant species. The median value and first and third quartiles in each group are also illustrated. Asterisks indicate statistical significance: *p < 0.05; **p < 0.01; ***p < 0.001; ns, *p* > 0.05.

An analysis of variance was performed on the normalized counts (Table 5). All four variables (biotype, infestation timepoint, host plant species, and host cultivar) significantly affected read counts, as did interactions of biotype with the other three variables. Tukey’s Honest Significant Difference test (Miller, 1981) showed that barley differed in observed OTUs from the other four species at adjusted *p*-value ≤0.01 (Table 6). Figure 6 demonstrates that barley supported a more diverse microflora. Moreover, all three barley cultivars yielded more OTUs than any other sampled cultivar. Specifically, read counts differed at *p* < 0.05 for nine pairwise comparisons involving biotype K on barley versus biotype K on the other four species and biotype E on any of the five species (Supplementary Table 1). The differences between day 8 and either of day 0 or day 2 were significant at *p* < 0.05, and day 8 had fewer OTUs (Supplementary Figure 1).

**Table 5:** ANOVA results based on observed read counts (P6T2).

Factor or interaction	Df	Sum Sq.	Mean Sq.	*F*-value	*p*-value	*R* ^2^	Sig
Biotype	1	187006.24	187006.24	7.74	0.00592	0.016	**
Host_Species	4	699281.34	174820.33	7.23	1.82 × 10^−5^	0.06	****
Cultivars	12	967006.90	80583.91	3.33	0.000191	0.084	***
Infestation_Time	3	398294.79	132764.93	5.49	0.0012	0.034	**
Biotype:Host_Species	4	305645.60	76411.40	3.16	0.0151	0.026	*
Biotype:Cultivars	12	1151239.67	95936.64	3.97	1.62 × 10^−5^	0.1	****
Biotype:Infestation_Time	3	534348.62	178116.21	7.37	0.000103	0.046	***
Host_Species:Infestation_Time	8	440873.87	55109.23	2.28	0.0234	0.038	*
Cultivars:Infestation_Time	24	870123.79	36255.16	1.5	0.0698	0.075	
Biotype:Host_Species:Infestation_Time	8	231841.42	28980.18	1.2	0.301	0.02	
Biotype:Cultivars:Infestation_Time	24	826247.37	34426.97	1.42	0.0984	0.071	
Residuals	205	4955004.67	24170.75				

Significance is *p < 0.05; **p < 0.01; ***p < 0.001; ****p < 0.0001.

**Table 6:** Results of Tukey’s Honest Significant Difference test, testing for differences in the observed OTUs.

Host pair	Host_Species difference	Lower bound	Upper bound	*p*. adj
B-A	138.17	45.27	231.07	0.00
R-A	5.75	−95.57	107.08	1.00
S-A	42.60	−45.78	130.98	0.67
W-A	16.57	−66.54	99.68	0.98
R-B	−132.42	−222.88	−41.96	0.00
S-B	−95.57	−171.25	−19.88	0.01
W-B	−121.60	−191.06	−52.15	0.00
S-R	36.85	−48.97	122.67	0.76
W-R	10.81	−69.56	91.19	1.00
W-S	−26.03	−89.32	37.26	0.79

Hosts are encoded A (*Aegilops*), B (barley), R (rye), S (sorghum), and W (wheat).

The mean Shannon diversity was 1.13 in biotype E and 1.19 in biotype K (Supplementary Figure 2). Mean Shannon diversity was 1.09 in *Aegilops*, 1.12 in rye, 1.14 in wheat, 1.16 in sorghum, and 1.30 in barley (Supplementary Figure 3). Three cultivars (Wintermalt barley, Post 90 barley, and TX2737 sorghum) accounted for the greater diversity in barley and sorghum (Supplementary Figure 4). Shannon diversity was also subjected to ANOVA (Supplementary Table 2), and the only significant differences involved the host plant species, the interaction of host plant species with aphid biotype and host cultivar, and the interaction of host plant species with aphid biotype and time. Tukey’s HSD test yielded six significant comparisons of Shannon diversity, again all involving biotype K on barley with biotype K on rye, wheat, or *Aegilops*, and biotype E on sorghum, wheat, or *Aegilops* (Supplementary Table 3 and Supplementary Figure 5). Barley, and only barley, differed significantly at *p* < 0.05 from the other host plant species in supported Shannon diversity (Supplementary Table 4). Shannon diversity did not vary significantly across days (Supplementary Table 5).

In ANOVA of Hill diversity (effective number of species, Supplementary Table 6), only host plant species and the interaction of host plant species with biotype and cultivar had significant effect. Once again, all significant pairwise comparisons with Tukey’s HSD involved biotype K on barley with biotype K on rye, wheat, or *Aegilops*, or biotype E on any host. Hill diversity was higher for biotype K than biotype E. For comparisons among species, the respective differences in mean significant Hill diversity versus barley were 1.84 for *Aegilops*, 1.74 for rye, 1.56 for wheat, and 1.36 for sorghum (Supplementary Table 7), and barley supported the highest diversity (4.9 versus 3.5 in sorghum, 3.3 in wheat, 3.1 in rye, and 3.0 in *Aegilops*). Cultivars Wintermalt, Post 90, and TX2737, yielded the highest Hill diversity (Supplementary Figure 6), and these were the cultivars with the highest fraction of non-*Buchnera* counts. There were no significant differences among infestation dates.

**Figure 7:**
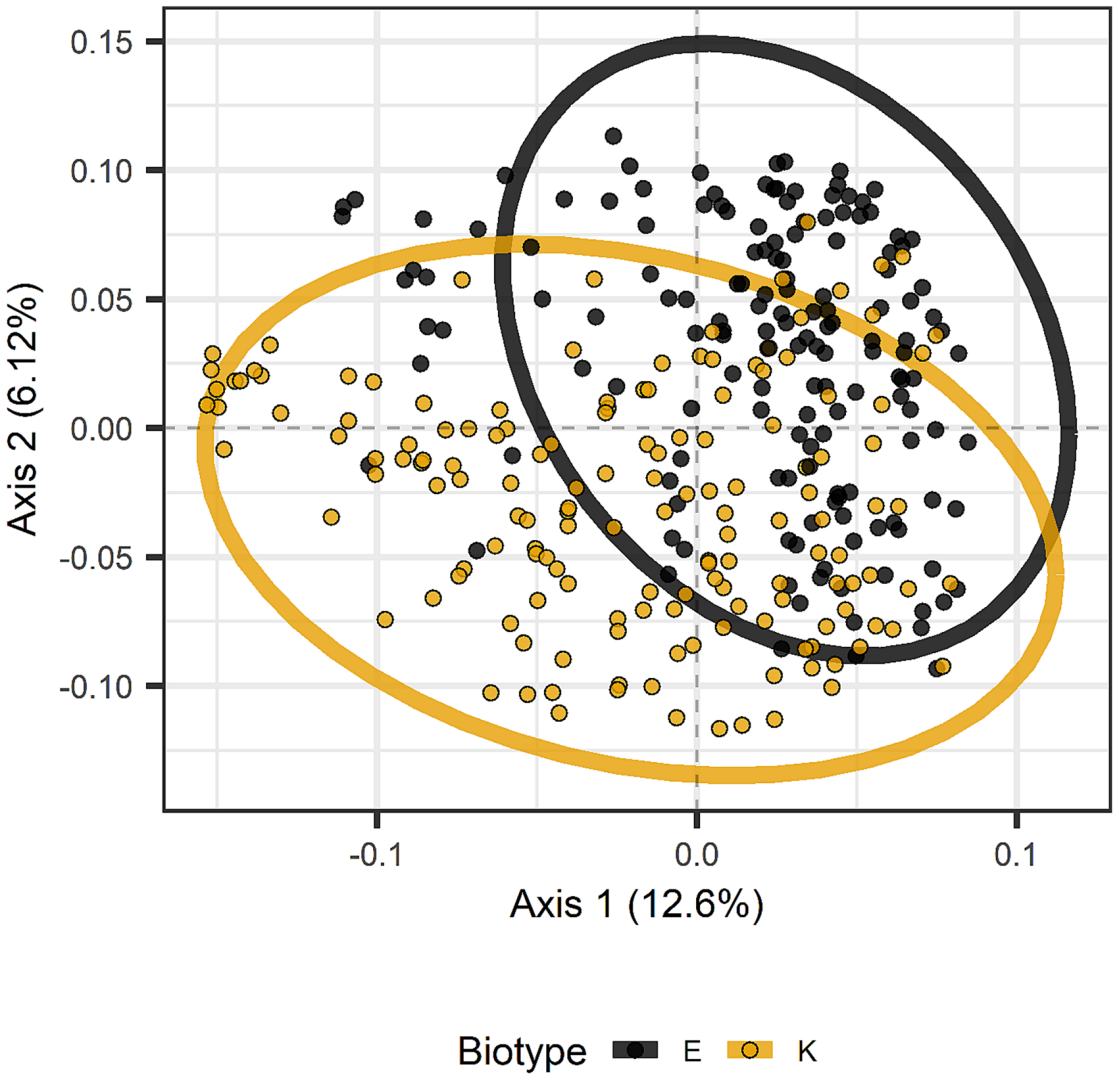
Principal coordinates analysis based on unweighted UniFrac distances distinguished by greenbug biotype. The distributions for biotypes E and K are mostly encircled.

Beta diversity (diversity among samples rather than within samples) was assessed with Adonis (Anderson, 2001) using weighted or unweighted UniFrac and Jaccard dissimilarities. In addition, pairwise Adonis tests were run for biotype, infestation date, and species. Results of the global Adonis tests appear in Table 7. Nine of eleven variables and interactions were significant with unweighted UniFrac, the only exceptions involving infestation date and host plant species (Table 7). With weighted UniFrac, only the interactions of biotype with host plant species or with host plant species and cultivars were significant. With Jaccard dissimilarity, the only significant variable or interaction was the interaction among biotype, host plant species, and host cultivar, although the interaction of biotype and host plant species was almost significant (*p* = 0.052) (Table 7). Pairwise Adonis with unweighted UniFrac gave significant differences between biotypes E and K, between barley and any of the other host plant species, and day 2 versus day 4 or 8. The difference between biotypes is illustrated in Figure 7, where the distributions overlap but mostly differ. Pairwise Adonis with weighted UniFrac also gave significant differences between biotypes E and K, along with significant difference between day 4 and the other days, but there were no significant differences among the host plant species. With Jaccard dissimilarity, pairwise Adonis detected only a significant interaction among biotype, host plant species, and cultivar.

**Table 7:** Results of global Adonis.

Factor or interaction	Df	Sums of Sq.	Mean Sqs.	*F*	*r* ^2^	Pr(>*F*)
Unweighted UniFrac						
Infestation_Time	3	1.78	0.59	2.74	0.02	0.001
Biotype	1	2.97	2.97	13.71	0.04	0.001
Host_Species	4	2.33	0.58	2.69	0.03	0.001
Infestation_Time:Biotype	3	1.37	0.46	2.11	0.02	0.001
Infestation_Time:Host_Species	8	2.10	0.26	1.21	0.03	0.016
Biotype:Host_Species	4	1.74	0.43	2.01	0.02	0.001
Host_Species:Cultivars	12	3.68	0.31	1.42	0.05	0.001
Infestation_Time:Host_Species:Cultivars	24	5.46	0.23	1.05	0.07	0.179
Infestation_Time:Biotype:Host_Species	8	1.83	0.23	1.05	0.02	0.273
Biotype:Host_Species:Cultivars	12	4.44	0.37	1.71	0.06	0.001
Infestation_Time:Biotype:Host_Species:Cultivars	24	5.88	0.24	1.13	0.08	0.013
Residuals	205	44.42	0.22		0.57	
Total	308	77.99			1.00	
Weighted UniFrac						
Infestation_Time	3	0.00	0.00	3.29	0.03	0.025
Biotype	1	0.00	0.00	1.92	0.01	0.147
Host_Species	4	0.00	0.00	1.17	0.01	0.310
Infestation_Time:Biotype	3	0.00	0.00	1.17	0.01	0.243
Infestation_Time:Host_Species	8	0.00	0.00	1.16	0.03	0.307
Biotype:Host_Species	4	0.01	0.00	3.41	0.04	0.005
Host_Species:Cultivars	12	0.01	0.00	1.40	0.05	0.098
Infestation_Time:Host_Species:Cultivars	24	0.01	0.00	1.06	0.07	0.362
Infestation_Time:Biotype:Host_Species	8	0.00	0.00	1.06	0.02	0.374
Biotype:Host_Species:Cultivars	12	0.01	0.00	1.67	0.06	0.030
Infestation_Time:Biotype:Host_Species:Cultivars	24	0.01	0.00	1.11	0.08	0.295
Residuals	205	0.08	0.00		0.59	
Total	308	0.14			1.00	
Jaccard						
Infestation_Time	3	0.01	0.00	0.72	0.01	0.519
Biotype	1	0.01	0.01	1.24	0.00	0.252
Host_Species	4	0.03	0.01	1.61	0.02	0.080
Infestation_Time:Biotype	3	0.01	0.00	0.55	0.01	0.646
Infestation_Time:Host_Species	8	0.04	0.00	0.89	0.02	0.563
Biotype:Host_Species	4	0.04	0.01	1.86	0.02	0.052
Host_Species:Cultivars	12	0.08	0.01	1.30	0.05	0.120
Infestation_Time:Host_Species:Cultivars	24	0.13	0.01	1.07	0.08	0.305
Infestation_Time:Biotype:Host_Species	8	0.05	0.01	1.30	0.03	0.186
Biotype:Host_Species:Cultivars	12	0.10	0.01	1.61	0.06	0.024
Infestation_Time:Biotype:Host_Species:Cultivars	24	0.12	0.01	1.02	0.07	0.452
Residuals	205	1.04	0.01		0.63	
Total	308	1.66			1.00	

The physiological activity of *Buchnera* versus the rest of the greenbug microbiome was inferred computationally with PICRUSt2 (Douglas et al., 2020), which output pairs of pathway and count related to the population and activity of each identified bacterial or archaeal taxon. Because of the high fraction of *Buchnera* reads, *Buchnera* counts exceeded other counts more than 10-fold. Rankings of pathways by count appear in Table 8 and Supplementary Table 8. Table 8 and Supplementary Table 8 have columns for all genera, *Buchnera* only, and all genera except *Buchnera*. Because of the abundance of *Buchnera*, the ranking of the most expressed pathways overall followed the ranking in *Buchnera*. The rankings indicated that *Buchnera* had lost 115 out of 421 pathways noted in other genera, including 112 of the 200 lowest-ranking by count and 85 of the 100 lowest-ranking by count. Thus *Buchnera* had selectively retained the more important pathways. Table 9 and Supplementary Table 9 contain comparative rankings of pathways ordered by activity over all genera except *Buchnera*. The most important pathway in the lumen bacteria was aerobic respiration utilizing cytochrome C, which releases hydrogen ions into the bacterial environment. This pathway ranked 175th in *Buchnera*, where acidifying the bacteriocyte cytoplasm is maladaptive. Earlier steps in respiration were not diminished; the tricarboxylic acid cycle ranked third in *Buchnera* and 14th elsewhere, while the non-oxidative part of the pentose phosphate pathway ranked fifth in *Buchnera* and 20th elsewhere. At least nine pathways in the synthesis and salvage of long-chain fatty acids were also diminished in *Buchnera*, including gondoate biosynthesis (60th in *Buchnera*, third elsewhere), mycolate biosynthesis (162nd in *Buchnera*, fourth elsewhere), cis-vaccenate biosynthesis (77th in *Buchnera*, 11th elsewhere), and fatty acid salvage (196th in *Buchnera*, 22nd elswhere). However, beta oxidation of fatty acids was increased (first in *Buchnera*, tenth elsewhere). Synthesis of most amino acids ranked similarly in *Buchnera* and elsewhere. An example was L-valine biosynthesis, ranked 12th in *Buchnera* and eighth elsewhere.

**Table 8:** Top 30 pathways identified by PICRUSt2.

Overall		Buchnera		All other genera	
Pathway	Count	Pathway	Count	Pathway	Count
Fatty acid beta-oxidation I	49990374.39	Fatty acid beta-oxidation I	47863170.30	Aerobic respiration I (cytochrome C)	4020259.41
Anhydromuropeptides recycling	43950267.23	Anhydromuropeptides recycling	42417511.74	Pyruvate fermentation to isobutanol (engineered)	2772569.39
TCA cycle I (prokaryotic)	43150337.13	TCA cycle I (prokaryotic)	41048308.93	Gondoate biosynthesis (anaerobic)	2763286.94
Glycogen degradation I (bacterial)	41334843.09	Glycogen degradation I (bacterial)	39880951.18	Mycolate biosynthesis	2430000.10
Pentose phosphate pathway (non-oxidative branch)	40209945.56	Pentose phosphate pathway (non-oxidative branch)	38368121.19	L-isoleucine biosynthesis II	2422960.26
L-isoleucine biosynthesis II	39849625.83	L-isoleucine biosynthesis II	37428656.17	Fatty acid elongation—saturated	2396467.81
Mixed acid fermentation	38199142.29	Mixed acid fermentation	36682065.34	L-valine biosynthesis	2313292.91
Pyruvate fermentation to isobutanol (engineered)	38100324.98	Superpathway of L-alanine biosynthesis	35986568.69	L-isoleucine biosynthesis I (from threonine)	2313292.91
L-valine biosynthesis	37781648.12	Peptidoglycan maturation (meso-diaminopimelate containing)	35935247.60	Oleate biosynthesis IV (anaerobic)	2258649.33
L-isoleucine biosynthesis I (from threonine)	37781648.12	TCA cycle VII (acetate-producers)	35921454.54	Fatty acid beta-oxidation I	2243395.81
Superpathway of L-alanine biosynthesis	37772950.94	Pyruvate fermentation to isobutanol (engineered)	35526994.20	Cis-vaccenate biosynthesis	2222135.39
TCA cycle VII (acetate-producers)	37632344.03	L-valine biosynthesis	35476963.41	Palmitoleate biosynthesis I (from (5Z)-dodec-5-enoate)	2194399.64
Peptidoglycan maturation (meso-diaminopimelate containing)	37561343.79	L-isoleucine biosynthesis I (from threonine)	35476963.41	Stearate biosynthesis II (bacteria and plants)	2148106.46
Superpathway of branched amino acid biosynthesis	36744146.58	Superpathway of branched amino acid biosynthesis	34714338.68	TCA cycle I (prokaryotic)	2140322.97
Sulfate reduction I (assimilatory)	35835117.59	Sulfate reduction I (assimilatory)	33815384.14	(5Z)-dodec-5-enoate biosynthesis	2139672.10
Superpathway of phospholipid biosynthesis I (bacteria)	34688494.38	Superpathway of phospholipid biosynthesis I (bacteria)	32695985.67	Superpathway of fatty acid biosynthesis initiation (*E. coli*)	2121814.72
Superpathway of adenosine nucleotides *de novo* biosynthesis I	34337541.32	Starch degradation V	32652900.69	Superpathway of L-serine and glycine biosynthesis I	2114370.67
L-isoleucine biosynthesis III	34249341.67	Superpathway of adenosine nucleotides *de novo* biosynthesis I	32550201.50	CDP-diacylglycerol biosynthesis I	2100599.12
Starch degradation V	34051427.17	L-isoleucine biosynthesis III	32366604.71	CDP-diacylglycerol biosynthesis II	2100599.12
Guanosine deoxyribonucleotides *de novo* biosynthesis II	33696318.85	Guanosine deoxyribonucleotides *de novo* biosynthesis II	31948746.12	Pentose phosphate pathway (non-oxidative branch)	2090619.61
Adenosine deoxyribonucleotides *de novo* biosynthesis II	33696318.85	Adenosine deoxyribonucleotides *de novo* biosynthesis II	31948746.12	Superpathway of branched amino acid biosynthesis	2055294.89
Superpathway of L-isoleucine biosynthesis I	33623938.23	Superpathway of polyamine biosynthesis I	31898110.50	Fatty acid salvage	1993050.79
Phosphatidylglycerol biosynthesis II (non-plastidic)	32704959.97	Hexitol fermentation to lactate, formate, ethanol and acetate	31862604.60	Superpathway of phospholipid biosynthesis I (bacteria)	1991151.37
Phosphatidylglycerol biosynthesis I (plastidic)	32704959.97	dTDP-N-acetylthomosamine biosynthesis	31861896.87	Superpathway of sulfate assimilation and cysteine biosynthesis	1965566.64
Superpathway of adenosine nucleotides *de novo* biosynthesis II	32681382.85	Superpathway of L-isoleucine biosynthesis I	31801573.14	L-isoleucine biosynthesis III	1920492.83
Superpathway of polyamine biosynthesis I	32545588.40	Superpathway of L-methionine biosynthesis (transsulfuration)	31406712.49	Urate biosynthesis/inosine 5′-phosphate degradation	1915638.53
L-lysine biosynthesis III	32515917.24	Superpathway of adenosine nucleotides *de novo* biosynthesis II	30965700.29	8-amino-7-oxononanoate biosynthesis I	1899244.13
Superpathway of L-methionine biosynthesis (transsulfuration)	32509176.89	Phosphatidylglycerol biosynthesis II (non-plastidic)	30824987.37	Sulfate reduction I (assimilatory)	1899170.77
dTDP-N-acetylthomosamine biosynthesis	32439110.11	Phosphatidylglycerol biosynthesis I (plastidic)	30824987.37	Peptidoglycan maturation (meso-diaminopimelate containing)	1893845.34
Superpathway of L-phenylalanine biosynthesis	32293766.33	L-lysine biosynthesis III	30802845.50	Superpathway of adenosine nucleotides *de novo* biosynthesis I	1886912.51

**Table 9:** Comparative ranking of pathways between *Buchnera* and other bacteria.

Pathway	Rank in others	Rank in *Buchnera*
Aerobic respiration I (cytochrome C)	1	175
Pyruvate fermentation to isobutanol (engineered)	2	11
Gondoate biosynthesis (anaerobic)	3	60
Mycolate biosynthesis	4	162
L-isoleucine biosynthesis II	5	6
Fatty acid elongation—saturated	6	61
L-isoleucine biosynthesis I (from threonine)	7	13
L-valine biosynthesis	8	12
Oleate biosynthesis IV (anaerobic)	9	54
Fatty acid beta-oxidation I	10	1
Cis-vaccenate biosynthesis	11	77
Palmitoleate biosynthesis I (from (5Z)-dodec-5-enoate)	12	71
Stearate biosynthesis II (bacteria and plants)	13	89
TCA cycle I (prokaryotic)	14	3
(5Z)-dodec-5-enoate biosynthesis	15	39
Superpathway of fatty acid biosynthesis initiation (*E. coli*)	16	59
Superpathway of L-serine and glycine biosynthesis I	17	104
CDP-diacylglycerol biosynthesis II	18	42
CDP-diacylglycerol biosynthesis I	19	41
Pentose phosphate pathway (non-oxidative branch)	20	5
Superpathway of branched amino acid biosynthesis	21	14
Fatty acid salvage	22	196
Superpathway of phospholipid biosynthesis I (bacteria)	23	16
Superpathway of sulfate assimilation and cysteine biosynthesis	24	38
L-isoleucine biosynthesis III	25	19
Urate biosynthesis/inosine 5′-phosphate degradation	26	48
8-amino-7-oxononanoate biosynthesis I	27	88
Sulfate reduction I (assimilatory)	28	15
Peptidoglycan maturation (meso-diaminopimelate containing)	29	9
Superpathway of adenosine nucleotides *de novo* biosynthesis I	30	18

## Discussion

A conventional study was conducted with the V5–V7 hypervariable region of the 16S rRNA gene in DNA isolated from intact greenbug aphids on 17 cultivars in five host plant species. There were four timepoints post infestation and two aphid biotypes defined by relative virulence on a set of wheat varieties. As in several other studies of 16S rRNA amplicons from aphid microbiomes, *Buchnera aphidicola* overwhelmingly dominated the read counts, ranging from 78.24 to 99.99% of the total. In *Rhopalosiphum padi*, *B. aphidicola* comprised 98.4% of the counts (Zepeda-Paulo et al., 2018) or 96.4% (Fakhour et al., 2018). In *Aphis glycines*, *B. aphidicola* comprised 70.3 to 93.5% of counts among geographic locations (Gallo-Franco et al., 2019). In *Aphis gossypii*, *B. aphidicola* was 2.9 to 74 times as counted as the second-place taxon (Ma et al., 2021). In *Melanaphis sacchari*, *B. aphidicola* comprised 90 to 99% of counts (Holt et al., 2020).

In all of these cases, including the current study, counts of *B. aphidicola* were inflated by its high copy number of 50 to 200 genomes per cell (Komaki and Ishikawa, 1999, 2000), versus one per cell in typical stationary-phase bacteria. Thus the populations of *B. aphidicola* and the more abundant gut bacteria like *Pseudomonas* might have been relatively similar. The count of *Buchnera* bacteroids per bacteriocyte also responds to environment (at least temperature; Neiers et al., 2021) and increases as the aphid ages. Another possible influence is unequally efficient amplification of various 16S rRNA loci by the degenerate forward primer used here, or unequal amplification based on the specific amplified sequences themselves. A few percent difference in amplification at each PCR cycle can mount up to several fold or more variation in read counts after 30 cycles. To demonstrate this, a simulation was conducted with 1,000 equally abundant taxa, 30 cycles, and amplification rates defined as 1 + (*m* – 0.5), where *m* is the mean of *N* = 20 to 50 random numbers in (0, 1). This resulted in the top 1% of taxa accounting for 11.5% of total amplicon counts with *N* = 50, 13.9% of total counts with *N* = 40, 17.8% with *N* = 30, and 24.8% with *N* = 20. The mean difference of amplification rates from 1.000 was 3.26% with *N* = 50, 3.65% with *N* = 40, 4.21% with *N* = 30, and 5.16% with *N* = 20.

The high fraction of *Buchnera* read counts generally detected in aphid microbiomes implies that the aphid gut lumen hosts relatively few bacteria. The plant sap from phloem is unlikely to harbor many bacteria in healthy plants, so the gut microflora must proliferate during transit through the digestive tract. How much it proliferates depends upon transit duration as well as antimicrobial substances, if any, ingested or secreted by the aphid. The interval between the start of feeding and the start of honeydew excretion is related to transit time, and this interval is measured in minutes for some aphid species. Its duration in greenbug seems not to have been measured in relation to environmental influences such as temperature and relative humidity.

The cause of the uneven distribution of non-*Buchnera* counts among treatments and replicates in Table 2 is not known, but the relative constancy of *Buchnera* counts favors a biological cause rather than differential PCR amplification. Possibly interactions among the microbes led to outbreaks of individual taxa in small groups of aphids, much like epidemics of disease. Also, the non-*Buchnera* sample size was very small in two biotype-timepoints with the highest fraction of *Buchnera* counts, and this increased the influence of sampling error. Thus it was stated in the Results that *Rhodanobacter* was abundant in biotype E on day 0 and biotype K on day 4, but it was stated earlier that *Rhodanobacter* was almost entirely restricted to a single biotype-timepoint (biotype K on day 4). This apparent contradiction resulted from the sum of non-*Buchnera* reads being 423 for biotype E on day 0 and 251,205 for biotype K on day 4. Non-*Buchnera* reads summed to 6,215 in biotype K on day 0 and exceeded 46,000 for the remaining combinations of biotype and timepoint.

Other beneficial symbionts (*Serratia symbiotica*, *Hamiltonella defensa*, *Regiella insecticola*, *Wolbachia*, *Arsenophonus* sp., *Candidatus Ishikawella capsulata*; Bansal et al., 2013; Gauthier et al., 2015; Jousselin et al., 2016; Fakhour et al., 2018; Enders et al., 2022) that have been proposed in other aphid species appeared to be absent in greenbug, since no OTUs mapped to them. We detected eight of the 15 other genera noted by Holt et al. (2020) in *Melanaphis sacchari*, and two (*Pseudomonas* and *Bacillus*) were abundant in our non-*Buchnera* counts. Among other genera that we found, Ma et al. (2021) reported *Acinetobacter*, *Flavobacterium*, and *Pantoea* in *Aphis gossypii*, and Enders et al. (2022) reported *Cutibacterium* and *Pantoea* from two *Aphis* species. Several other genera (*Rhodanobacter*, *Chryseobacterium*, *Luteibacter*, and *Pedobacter*) that were relatively common in the current study appear not to have been reported previously in aphid microfloras.

Microbial contamination of labware and PCR reagents can lead to spurious detection of bacterial taxa that are common in the environment. Genera such as *Escherichia*, *Pelomonas*, and *Methylobacterium*, have appeared in reads of PCR product from blank samples (Stinson et al., 2018). Thus, it is not apparent which low-frequency reads came from the reagents and which if any came from low frequencies of the same genera in DNA samples.

It is obvious from the diversity in reported microbes in aphids that aphid genotype and diet affect the microbiome in aphids. He et al. (2021) provide a detailed account of the effect of diet (Yun et al., 2014). They moved a clone of peach aphids (*Myzus persicae*) from Chinese cabbage to eggplant, tobacco, or pepper. On cabbage, the top-ranking genera were *Buchnera* (67.7% of counts), *Acinetobacter* (7.8%), and *Allobaculum* (5.8%); *Pseudomonas* was only 0.6%. On eggplant, the top four were *Pseudomonas* (69.4%), *Buchnera* (12.5%), *Ralstonia* (2.1%), and *Burkholderia* (1.7%). On tobacco, the top genera were *Pseudomonas* (62.2%), *Buchnera* (14.6%), *Ralstonia* (3.0%), and *Burkholderia* (2.1%). On pepper the top genera shifted to *Buchnera* (25.5%), *Pseudomonas* (11.7%), *Ralstonia* (8.4%), *Burkholderia* (5.0%), unassigned Enterobacteriaceae (3.2%), *Acinetobacter* (2.3%), and *Stenotrophomonas* (1.7%). Thus, eggplant and tobacco greatly favored *Pseudomonas* and somewhat suppressed *Acinetobacter*, and there was a rebalancing of total counts between the gut and the bacteriocytes. The authors did not consider the high ploidy of *Buchnera* in regard to its counts.

With 22 significantly affected genera (Table 3), greenbug biotype was more important than host species, which affected seven genera, or collection time, which affected only two genera. Accordingly, biotype K on barley had the most diverse bacterial communities that were least overwhelmed by the abundance of *Buchnera*. The eight-day study interval was insufficient time for the aphids to kill the host plant or produce alate morphs, as would be expected at saturation. Resistance genes, which reduced the size of individual greenbugs and reduced their population, had no consistent effect on the microbiome. Either none of the resistance genes affected the microbiome, or they varied in mode of action and thus did not produce a consistent change in microbial abundance. In concept, biotype could affect interactions of microbes with greenbug immunity, the efficiency with which the greenbugs absorbed nutrients from the gut, or the flow of amino acids into and out of the *Buchnera* bacteroids. However, another plausible mechanism is historical. Greenbug nymphs receive *Buchnera* from their mother during embryogenesis and acquire lumen bacteria from the environment after birth. However, in an established aphid population, the environment contains honeydew from older aphids, enabling an indirect sort of vertical transmission of gut bacteria from the founders of the population. The role of population history could be tested by moving populations of each biotype back and forth to areas where the other biotype has fed and defecated, and seeing if the microbiomes converge. Molecular genetic markers could be used to control for biotype replacement within the populations.

Microbiome studies in aphids frequently cite the role of *Buchnera* in providing essential amino acids and remedying the amino acid imbalance in plant sap. However, *Buchnera* merely redistributes nitrogen among amino acids; it does not increase the total nitrogen supply. Our study encountered low levels of five genera of typically nitrogen-fixing bacteria (*Rhizobium*, *Bradyrhizobium*, *Azospirillum*, *Azospira*, and *Azohydromonas*) in the greenbug microbiome without revealing whether they were in the gut or on the phylloplane, which was inadvertently sampled when the greenbugs were collected. This raises the question of whether appreciable nitrogen fixation occurs in the gut. The PICRUSt2 analysis did not reveal *nif* genes, indicating that the aphid microbiome does not fix nitrogen. Direct measurement of nitrogen fixation or acetylene reduction might be warranted to answer this question. Bacterial nitrogen fixation has been documented in wood-feeding termites of the family Kalotermitidae (Mullins and Su, 2023).

Despite the established role of *Buchnera* in supplying essential amino acids that are deficient in phloem sap, the PICRUSt2 results did not indicate increased expression of pathways to produce these amino acids relative to the rest of the microbiome. Nevertheless, there is experimental evidence that synthesis of various amino acids is not feedback inhibited or repressed in *Buchnera* (Wilcox et al., 2003). The most obvious difference that set *Buchnera* apart was the suppression of electron transport, which releases hydrogen ions to the exterior of the bacterial cell. This suppression is likely an adaptation to life within bacteriocyte cytoplasm, where acidification would be harmful. The diminution of gondoic, mycolic, and cis-vaccenic acids affects the fluidity and thus permeability of *Buchnera* cell membranes in seemingly contradictory ways. Diminishing mycolic acid expectedly increases fluidity and permeability, but decreasing gondoic and cis-vaccenic acids expectedly decreases fluidity (Marrakchi et al., 2014). Increased permeability would aid export of amino acids from the bacteroids to the bacteriocytes. Conclusions about the role of *Buchnera* are affected by the great variation among strains in genome size (from 412 to 646 kb) and gene content (354 to 587) (Chong et al., 2019); the strain in *Schizaphis graminum* is near the upper end of these ranges and expectedly retains more of the functionality of free-living bacteria.

## Data Availability

The datasets presented in this study can be found in online repositories. The names of the repository/repositories and accession number(s) can be found at: https://www.ncbi.nlm.nih.gov/, PRJNA1170337. Scripts can be found at https://github.com/cfcrane/greenbug-16S-microbiome.

## Author’s note

Mention of a commercial or proprietary product does not constitute an endorsement by the US Federal Government.

## Supplementary material

SUPPLEMENTARY FIGURE 1 Mean zero-radius OTUs observed across infestation times. Vertical line segments at each point denote a 95% confidence interval for the infestation time. Horizontal dashed line shows the mean value across the full dataset.

SUPPLEMENTARY FIGURE 2 Mean Shannon diversity across biotypes. Vertical line segments at each point denote a 95% confidence interval for the biotype. Horizontal dashed line shows the mean value across the full dataset.

SUPPLEMENTARY FIGURE 3 Mean Shannon diversity across host plant species. Vertical line segments at each point denote a 95% confidence interval for the host plant species. Horizontal dashed line shows the mean value across the full dataset.

SUPPLEMENTARY FIGURE 4 Mean Shannon diversity across cultivars. Vertical line bars at each point denote a 95% confidence interval for the cultivars. Horizontal dashed line shows the mean value across the full dataset.

SUPPLEMENTARY FIGURE 5 Shannon diversity within the total microbiome data, grouped and colored by cultivars, facetted by biotype and host plant species. The median value and first and third quartiles in each group are also illustrated.

SUPPLEMENTARY FIGURE 6 Mean Hill1 diversity across cultivars. Vertical line segments at each point denote a 95% confidence interval for the cultivars. Horizontal dashed line shows the mean value across the full dataset.
