# Supplementary material for: Biotype and host relatedness influence the composition of bacterial microbiomes in Schizaphis graminum aphids
Source: Front Microbiol. 2025 Jul 30;16:1614492. doi: 10.3389/fmicb.2025.1614492 (PMC12345607; doi:10.3389/fmicb.2025.1614492)
Supplement: Supplementary file 7 [file Table_1.docx]

Supplemental Table 1 Results of Tukey’s HSD, testing for differences in the observed zero-radius OTUs.

| Comparison | Biotype.Host_Species.diff | lwr | upr | p.adj |
| --- | --- | --- | --- | --- |
| K:A-E:A | 45.45 | -125.39 | 216.29 | 1.00 |
| E:B-E:A | 86.51 | -62.18 | 235.21 | 0.70 |
| K:B-E:A | 235.21 | 86.51 | 383.91 | 0.00 |
| E:R-E:A | 66.26 | -96.63 | 229.14 | 0.95 |
| K:R-E:A | -9.36 | -172.25 | 153.52 | 1.00 |
| E:S-E:A | 23.67 | -117.39 | 164.74 | 1.00 |
| K:S-E:A | 106.91 | -34.15 | 247.98 | 0.32 |
| E:W-E:A | 35.90 | -96.22 | 168.02 | 1.00 |
| K:W-E:A | 42.62 | -89.50 | 174.74 | 0.99 |
| E:B-K:A | 41.06 | -116.30 | 198.43 | 1.00 |
| K:B-K:A | 189.76 | 32.39 | 347.12 | 0.01 |
| E:R-K:A | 20.80 | -150.04 | 191.64 | 1.00 |
| K:R-K:A | -54.82 | -225.66 | 116.02 | 0.99 |
| E:S-K:A | -21.78 | -171.96 | 128.39 | 1.00 |
| K:S-K:A | 61.46 | -88.71 | 211.64 | 0.95 |
| E:W-K:A | -9.55 | -151.35 | 132.26 | 1.00 |
| K:W-K:A | -2.84 | -144.64 | 138.97 | 1.00 |
| K:B-E:B | 148.70 | 15.70 | 281.69 | 0.02 |
| E:R-E:B | -20.26 | -168.96 | 128.44 | 1.00 |
| K:R-E:B | -95.88 | -244.57 | 52.82 | 0.56 |
| E:S-E:B | -62.84 | -187.25 | 61.57 | 0.84 |
| K:S-E:B | 20.40 | -104.01 | 144.81 | 1.00 |
| E:W-E:B | -50.61 | -164.77 | 63.55 | 0.92 |
| K:W-E:B | -43.90 | -158.06 | 70.27 | 0.97 |
| E:R-K:B | -168.96 | -317.65 | -20.26 | 0.01 |
| K:R-K:B | -244.57 | -393.27 | -95.88 | 0.00 |
| E:S-K:B | -211.54 | -335.95 | -87.13 | 0.00 |
| K:S-K:B | -128.30 | -252.70 | -3.89 | 0.04 |
| E:W-K:B | -199.31 | -313.47 | -85.14 | 0.00 |
| K:W-K:B | -192.59 | -306.76 | -78.43 | 0.00 |
| K:R-E:R | -75.62 | -238.51 | 87.27 | 0.90 |
| E:S-E:R | -42.58 | -183.65 | 98.48 | 0.99 |
| K:S-E:R | 40.66 | -100.41 | 181.72 | 1.00 |
| E:W-E:R | -30.35 | -162.47 | 101.77 | 1.00 |
| K:W-E:R | -23.64 | -155.76 | 108.48 | 1.00 |
| E:S-K:R | 33.04 | -108.03 | 174.10 | 1.00 |
| K:S-K:R | 116.28 | -24.79 | 257.34 | 0.21 |
| E:W-K:R | 45.27 | -86.85 | 177.39 | 0.98 |
| K:W-K:R | 51.98 | -80.14 | 184.10 | 0.96 |
| K:S-E:S | 83.24 | -31.94 | 198.42 | 0.39 |
| E:W-E:S | 12.23 | -91.80 | 116.26 | 1.00 |
| K:W-E:S | 18.94 | -85.09 | 122.98 | 1.00 |
| E:W-K:S | -71.01 | -175.04 | 33.02 | 0.47 |
| K:W-K:S | -64.30 | -168.33 | 39.73 | 0.62 |
| K:W-E:W | 6.71 | -84.82 | 98.25 | 1.00 |
