# Supplementary material for: Biotype and host relatedness influence the composition of bacterial microbiomes in Schizaphis graminum aphids
Source: Front Microbiol. 2025 Jul 30;16:1614492. doi: 10.3389/fmicb.2025.1614492 (PMC12345607; doi:10.3389/fmicb.2025.1614492)
Supplement: Supplementary file 8 [file Table_2.docx]

Supplemental Table 2. Results of ANOVA, testing for differences in Hill1 diversity among groups.

| Factor or Interaction | Df | Sum Sq | Mean Sq | F value | P value | R2 | Sig |
| --- | --- | --- | --- | --- | --- | --- | --- |
| Biotype | 1 | 30.54 | 30.54 | 3.4 | 0.0668 | 0.01 | . |
| Host_Species | 4 | 115.60 | 28.90 | 3.21 | 0.0139 | 0.038 | * |
| Infestation_Time | 3 | 0.98 | 0.33 | 0.04 | 0.991 | 0 |  |
| Biotype:Host_Species | 4 | 86.07 | 21.52 | 2.39 | 0.0519 | 0.028 | . |
| Biotype:Infestation_Time | 3 | 8.46 | 2.82 | 0.31 | 0.816 | 0.003 |  |
| Host_Species:Cultivars | 12 | 163.26 | 13.61 | 1.51 | 0.122 | 0.053 |  |
| Host_Species:Infestation_Time | 8 | 74.89 | 9.36 | 1.04 | 0.407 | 0.024 |  |
| Biotype:Host_Species:Cultivars | 12 | 218.60 | 18.22 | 2.03 | 0.0236 | 0.071 | * |
| Biotype:Host_Species:Infestation_Time | 8 | 135.51 | 16.94 | 1.88 | 0.0643 | 0.044 | . |
| Host_Species:Cultivars:Infestation_Time | 24 | 218.10 | 9.09 | 1.01 | 0.455 | 0.071 |  |
| Biotype:Host_Species:Cultivars:Infestation_Time | 24 | 182.16 | 7.59 | 0.84 | 0.678 | 0.059 |  |
| Residuals | 205 | 1844.10 | 9.00 |  |  |  |  |
