# Supplementary material for: Biotype and host relatedness influence the composition of bacterial microbiomes in Schizaphis graminum aphids
Source: Front Microbiol. 2025 Jul 30;16:1614492. doi: 10.3389/fmicb.2025.1614492 (PMC12345607; doi:10.3389/fmicb.2025.1614492)
Supplement: Supplementary file 9 [file Table_3.docx]

Supplemental Table 3. Results of Tukey’s HSD, testing for differences in Shannon diversity.

| Comparison | Biotype.Host_Species.diff | lwr | upr | p.adj |
| --- | --- | --- | --- | --- |
| K:A-E:A | 0.06 | -0.27 | 0.39 | 1.00 |
| E:B-E:A | 0.13 | -0.16 | 0.41 | 0.92 |
| K:B-E:A | 0.36 | 0.07 | 0.64 | 0.00 |
| E:R-E:A | 0.09 | -0.22 | 0.40 | 1.00 |
| K:R-E:A | 0.03 | -0.28 | 0.34 | 1.00 |
| E:S-E:A | 0.05 | -0.22 | 0.32 | 1.00 |
| K:S-E:A | 0.14 | -0.13 | 0.41 | 0.81 |
| E:W-E:A | 0.08 | -0.17 | 0.34 | 0.99 |
| K:W-E:A | 0.08 | -0.17 | 0.33 | 0.99 |
| E:B-K:A | 0.07 | -0.23 | 0.37 | 1.00 |
| K:B-K:A | 0.30 | -0.00 | 0.60 | 0.05 |
| E:R-K:A | 0.03 | -0.30 | 0.36 | 1.00 |
| K:R-K:A | -0.03 | -0.36 | 0.30 | 1.00 |
| E:S-K:A | -0.01 | -0.30 | 0.28 | 1.00 |
| K:S-K:A | 0.08 | -0.21 | 0.37 | 1.00 |
| E:W-K:A | 0.02 | -0.25 | 0.30 | 1.00 |
| K:W-K:A | 0.02 | -0.25 | 0.29 | 1.00 |
| K:B-E:B | 0.23 | -0.02 | 0.49 | 0.11 |
| E:R-E:B | -0.04 | -0.32 | 0.25 | 1.00 |
| K:R-E:B | -0.10 | -0.38 | 0.19 | 0.99 |
| E:S-E:B | -0.08 | -0.31 | 0.16 | 0.99 |
| K:S-E:B | 0.01 | -0.22 | 0.25 | 1.00 |
| E:W-E:B | -0.04 | -0.26 | 0.18 | 1.00 |
| K:W-E:B | -0.05 | -0.27 | 0.17 | 1.00 |
| E:R-K:B | -0.27 | -0.56 | 0.01 | 0.08 |
| K:R-K:B | -0.33 | -0.61 | -0.04 | 0.01 |
| E:S-K:B | -0.31 | -0.55 | -0.07 | 0.00 |
| K:S-K:B | -0.22 | -0.46 | 0.02 | 0.10 |
| E:W-K:B | -0.28 | -0.50 | -0.06 | 0.00 |
| K:W-K:B | -0.28 | -0.50 | -0.06 | 0.00 |
| K:R-E:R | -0.06 | -0.37 | 0.25 | 1.00 |
| E:S-E:R | -0.04 | -0.31 | 0.23 | 1.00 |
| K:S-E:R | 0.05 | -0.22 | 0.32 | 1.00 |
| E:W-E:R | -0.00 | -0.26 | 0.25 | 1.00 |
| K:W-E:R | -0.01 | -0.26 | 0.24 | 1.00 |
| E:S-K:R | 0.02 | -0.25 | 0.29 | 1.00 |
| K:S-K:R | 0.11 | -0.16 | 0.38 | 0.95 |
| E:W-K:R | 0.05 | -0.20 | 0.31 | 1.00 |
| K:W-K:R | 0.05 | -0.21 | 0.30 | 1.00 |
| K:S-E:S | 0.09 | -0.13 | 0.31 | 0.95 |
| E:W-E:S | 0.03 | -0.17 | 0.23 | 1.00 |
| K:W-E:S | 0.03 | -0.17 | 0.23 | 1.00 |
| E:W-K:S | -0.06 | -0.26 | 0.14 | 1.00 |
| K:W-K:S | -0.06 | -0.26 | 0.14 | 0.99 |
| K:W-E:W | -0.01 | -0.18 | 0.17 | 1.00 |
