# Supplementary material for: Biotype and host relatedness influence the composition of bacterial microbiomes in Schizaphis graminum aphids
Source: Front Microbiol. 2025 Jul 30;16:1614492. doi: 10.3389/fmicb.2025.1614492 (PMC12345607; doi:10.3389/fmicb.2025.1614492)
Supplement: Supplementary file 10 [file Table_4.docx]

Supplemental Table 4. Results of Tukey’s HSD test, testing for differences in the Shannon diversity.

| Species Pair | Host_Species.diff | lwr | upr | p.adj |
| --- | --- | --- | --- | --- |
| B-A | 0.21 | 0.04 | 0.39 | 0.01 |
| R-A | 0.03 | -0.16 | 0.22 | 0.99 |
| S-A | 0.07 | -0.10 | 0.24 | 0.82 |
| W-A | 0.05 | -0.11 | 0.21 | 0.90 |
| R-B | -0.18 | -0.36 | -0.01 | 0.03 |
| S-B | -0.15 | -0.29 | -0.00 | 0.05 |
| W-B | -0.16 | -0.30 | -0.03 | 0.01 |
| S-R | 0.04 | -0.13 | 0.20 | 0.97 |
| W-R | 0.02 | -0.13 | 0.18 | 1.00 |
| W-S | -0.02 | -0.14 | 0.11 | 1.00 |
