# Supplementary material for: Biotype and host relatedness influence the composition of bacterial microbiomes in Schizaphis graminum aphids
Source: Front Microbiol. 2025 Jul 30;16:1614492. doi: 10.3389/fmicb.2025.1614492 (PMC12345607; doi:10.3389/fmicb.2025.1614492)
Supplement: Supplementary file 11 [file Table_5.docx]

Supplemental Table 5. Results of Tukey’s HSD, testing for differences in the Shannon diversity over time.

| Days | Infestation_Time.diff | lwr | upr | p.adj |
| --- | --- | --- | --- | --- |
| 2-0 | 0.02 | -0.30 | 0.34 | 1.00 |
| 4-0 | 0.04 | -0.28 | 0.35 | 0.99 |
| 8-0 | -0.03 | -0.34 | 0.29 | 1.00 |
| 4-2 | 0.02 | -0.09 | 0.13 | 0.97 |
| 8-2 | -0.04 | -0.15 | 0.06 | 0.72 |
| 8-4 | -0.06 | -0.17 | 0.05 | 0.44 |
