# Supplementary material for: Biotype and host relatedness influence the composition of bacterial microbiomes in Schizaphis graminum aphids
Source: Front Microbiol. 2025 Jul 30;16:1614492. doi: 10.3389/fmicb.2025.1614492 (PMC12345607; doi:10.3389/fmicb.2025.1614492)
Supplement: Supplementary file 13 [file Table_7.docx]

Supplemental Table 7. Results of Tukey’s HSD, testing for differences in Hill1 Diversity.

| Species Pair | Host_Species.diff | lwr | upr | p.adj |
| --- | --- | --- | --- | --- |
| B-A | 1.84 | 0.01 | 3.66 | 0.05 |
| R-A | 0.10 | -1.89 | 2.09 | 1.00 |
| S-A | 0.48 | -1.26 | 2.21 | 0.94 |
| W-A | 0.28 | -1.35 | 1.91 | 0.99 |
| R-B | -1.74 | -3.51 | 0.04 | 0.06 |
| S-B | -1.36 | -2.85 | 0.13 | 0.09 |
| W-B | -1.56 | -2.92 | -0.20 | 0.02 |
| S-R | 0.37 | -1.31 | 2.06 | 0.97 |
| W-R | 0.17 | -1.40 | 1.75 | 1.00 |
| W-S | -0.20 | -1.44 | 1.04 | 0.99 |
